# Role of SCAP in regulation of pancreatic homeostasis, pancreatitis, and tumorigenesis

**DOI:** 10.1038/s41388-026-03784-y

**Published:** 2026-04-15

**Authors:** Anna C. Lilly, Valerii A. Pavlov, Shabnam Pirestani, Adam Chatoff, Kathy Q. Cai, Edna Cukierman, Nathaniel W. Snyder, Igor Astsaturov, Erica A. Golemis

**Affiliations:** 1 Program in Cancer Signaling and Microenvironment, Fox Chase Cancer Center, Philadelphia, PA USA; 2 Molecular & Cell Biology & Genetics (MCBG) Program, Drexel University College of Medicine, Philadelphia, PA USA; 3 Department of Allergy and Immunology, Cincinnati Children’s Hospital, Cincinnati, OH USA; 4 Aging + Cardiovascular Discovery Center, Lewis Katz School of Medicine, Temple University, Philadelphia, PA USA; 5 Marvin & Concetta Greenberg Pancreatic Cancer Institute, Fox Chase Cancer Center, Philadelphia, PA USA; 6 Department of Cancer and Cellular Biology, Lewis Katz School of Medicine, Temple University, Philadelphia, PA USA

**Keywords:** Pancreatic cancer, Cancer models, Differentiation

## Abstract

Levels of pancreatic ductal adenocarcinoma (PDAC) are increasing, with epidemiological studies nominating obesity, altered cholesterol metabolism, and elevated lipids as risk factors. In prior studies, we determined that elevated expression of sterol regulatory element binding protein 2 (SREBP2), a transcription factor directing lipid biosynthesis, promoted epithelial-mesenchymal transition and aggressive tumorigenesis in the KPC (*LSL-Kras*^*G12D*^*;Trp53*^*f/f*^*;Pdx1-Cre*) mouse model of PDAC. We analyzed the consequences of deleting SCAP, a scaffolding protein required for SREBP activation, in KPC mice. Unexpectedly tumorigenesis in KPCS mice was significantly accelerated, with a preponderance of sarcomatoid carcinomas. To better understand SCAP action, we analyzed loss of pancreatic SCAP in isolation in *Scap*^*Δpanc*^ *(Pdx1-Cre;Scap*^*f/f*^) mice. Pancreata of *Scap*^*Δpanc*^ mice had rapid progressive loss of acinar cells, acinar-ductal metaplasia (ADM), infiltration of adipose cells, increased fibrosis, and infiltration of immune cells, indicative of chronic pancreatitis. Single cell RNA sequencing indicated that loss of SCAP suppressed SREBP-dependent transcriptional programs in endocrine and exocrine precursors, but was associated with enhanced SREBP2 activity in fibroblastic populations, compatible with formation of a pro-tumorigenic tumor microenvironment. Together, these results implicate lipid metabolism via SCAP-SREBP signaling as an important metabolic regulator of acinar-ductal differentiation and pancreatic carcinogenesis.

## Introduction

Pancreatic ductal adenocarcinoma (PDAC) is the most common cancer of the pancreas, with pancreatic cancer affecting 67,440 individuals in the United States in 2025 [1]. Rates of PDAC have been rising in the general population, with the increase of associated risk factors including obesity, accumulation of visceral fat, diabetes, and chronic pancreatitis [2]. A common feature of many of these risk factors is dysregulated lipid metabolism. A core mechanism governing lipid metabolism is the SCAP-SREBP1/2 signaling axis.

Under physiological growth conditions, transient induction of the SREBP transcription factors (SREBP1a, SREBP1c, and SREBP2) induces genes involved in synthesis of fatty acids and cholesterol so as to support regulated cell growth and replacement [3]. Full-length SREBP proteins are typically retained at the ER based on interaction with an ER-associated transmembrane scaffolding protein known as SREBP cleavage activating protein (SCAP) [4]. Prior to activation of the pathway, SCAP interacts with a second protein, insulin-induced gene (INSIG), maintaining the SCAP-SREBPs complex in an inactive form [4, 5]. Upstream signals including elevated glucose disrupt the INSIG interaction with SCAP-SREBPs, freeing the SCAP-SREBP complex to translocate to the Golgi, where SREBPs are cleaved by the proteases S1P and S2P. This cleavage releases the N-terminal SREBP fragment, which translocates to the nucleus and activates transcription of genes including HMG-CoA synthase (HMGCS1), fatty acid synthase (FAS), HMG-CoA reductase (HMGCR), and others, leading to production of cholesterol and fatty acids. As levels of these lipids rise, cholesterol binds to SCAP and causes it to associate with INSIG [6, 7], turning off the cycle.

In past work, we reported that SREBP1 induced as a result of cholesterol depletion subsequently bound to the promoter of TGFβ1, a secreted cytokine which in turn induced an autocrine TGFBR-SMAD2/3 signaling cascade in PDAC cells [8]. This caused an epithelial-mesenchymal transition (EMT), and resulted in tumors with the more lethal basal subtype versus the classic more epithelial subtype in a KRAS mutated, P53-deficient mouse model (KPC mice) [8]. These results aligned with findings by others that elevated SREBP activity can contribute to other forms of gastrointestinal tumors [9]. Importantly, analysis of patient specimens also suggested a correlation between use of cholesterol-lowering statins, and a tendency toward EMT and hallmarks of aggressive disease in patient PDAC specimens [8]. These results raised the possibility that suppression of pancreatic SREBP signaling may provide a useful strategy to prevent more aggressive forms of PDAC but emphasized the need to investigate underlying signaling mechanisms.

To better understand the requirement for SREBP in the pancreas, we have now analyzed a mouse model in which *PDX1-*Cre causes loss of SCAP, and therefore loss of SREBP activation, in KPC mice. Unexpectedly, this resulted in elevated acinar-ductal metaplasia (ADM), accelerated tumor formation, shortened survival, and a conversion to more mesenchymal, sarcomatoid tumors. To gain insight into underlying mechanisms, we analyzed in detail the phenotypes associated with SCAP loss during pancreatic development, in the absence of introducing tumor driver mutations. In SCAP deficient pancreata, a rapid post-natal loss of exocrine acinar cells was accompanied by extensive replacement of the tissue mass with adipose cells, accompanied by immune cell infiltration, fibrosis, and ADM. Intriguingly, extensive mechanistic analysis of pancreata and PDAC tumors by single cell sequencing indicated loss of SCAP triggers reactive signaling between exocrine and fibroblastic cells that together create a pro-tumorigenic microenvironment.

## Methods

Additional details are provided as Supplementary Methods.

### Mouse models

The analysis used *Pdx1-Cre* mice ([10], Jax#014647, RRID:IMSR_JAX:014647) and *Scap*^*f/f*^ mice ([11], Jax#004162, RRID:IMSR_JAX:004162) obtained from Jackson laboratory (Bar Harbor, ME). *Ai9* (*B6.Cg-Gt(ROSA)26Sortm9(CAG-tdTomato)Hze/J*) mice ([12], Jax#007909, RRID:IMSR_JAX:007909) were used for lineage-tracing experiments. *KPC* (*LSL-Kras*^*G12D*^*;Trp53*^*f/f*^*;Pdx1-Cre*) mice [8] were used as a model for pancreatic ductal adenocarcinoma. Genotypes were confirmed by PCR using published primer sets established for each strain (Supplementary Table S1). All mice were bred and maintained under defined-flora, pathogen-free conditions at the AAALAC-approved Laboratory Animal Facility at Fox Chase Cancer Center. Age-matched mice of both sexes, equally distributed, were used for experiments unless specified otherwise. All mice were observed and weighed weekly throughout the time of experiments. Mice with AALAC-specified signs of distress or weight loss of more than 10% were euthanized by CO_2_ inhalation followed cervical dislocation per the Institutional Animal Cancer and Use Committee (IACUC) guidelines at Fox Chase Cancer Center, Philadelphia, PA.

### Analysis of pancreatic tissue

Pancreata were typically fixed by standard methods described in the Supplementary Methods, and imaging performed following H&E staining, or processing of specimens for immunohistochemistry or immunofluorescence using standard approaches. All antibodies in the study are listed in Supplementary Table S2.

### Single cell and single nuclei RNA isolation and sequencing

Specimens were processed for single cell sequencing analysis using standard techniques described in the Supplementary Methods, and per instructions from manufacters. Libraries were produced using reagents from 10X Genomics (Pleasanton, CA), and processed by Novogene (Sacramento, CA).

### Single cell sequencing analysis

Data was processed and analysis performed using standard tools: Cell Ranger 7.1.0 and 8.0.1 (10x Genomics) and the Seurat analytic pipeline (version 5.2.1, RRID:SCR_016341), using previously described methods [13]. GSEA analysis was performed using Hallmark gene sets [14, 15] for benchmarking. The complete script is available in a Github repository (https://github.com/lillya349/Scap-project). Additional analysis was performed using Ingenuity pathway analysis (Qiagen, Germantown, MD, RRID:SCR_008653), and Jensen Disease analysis [16], based on an input gene list developed by Enrichr [17, 18]. Pseudotime analysis was performed using standard approaches [19–22].

### Analysis of cell lines

AR42J cells were generously provided by Dr. Mark Hellmich of the University of Texas Medical Branch, and their identity was confirmed by STR profiling. SCAP depletion by siRNA, qRT-PCR, and Western analysis were performed by standard means described in the Supplementary Methods. Quantification was done using Fiji (PMID: 22743772, version 2.14.0, National Institute of Health, Bethesda, MD), and all values were normalized to relative expression of GAPDH (Cat#97166, Cell Signaling, Danvers, MA). Primers used are listed in Supplementary Table S1.

### Lipid extraction and Liquid chromatography-high resolution mass spectrometry (LC-HRMS) for lipids

Lipidomic analysis was performed on pancreata dissected from 2-week-old *Scap*^*f/f*^ and *Scap*^*Δpanc*^, using LC-HRMS as previously described [23]. Untargeted analysis and targeted peak integration was conducted using LipidsSearch 4.2 (Thermo Fisher Scientific, Waltham, MA) as described [24].

### Statistical analysis

Unpaired *t* tests were used for comparisons unless otherwise noted. Analysis was performed using GraphPad Prism 8.0.1 (GraphPad Software, San Diego, CA).

## Results

### Loss of SCAP accelerates KRAS/TP53-induced pancreatic tumor formation

**Fig. 1:**
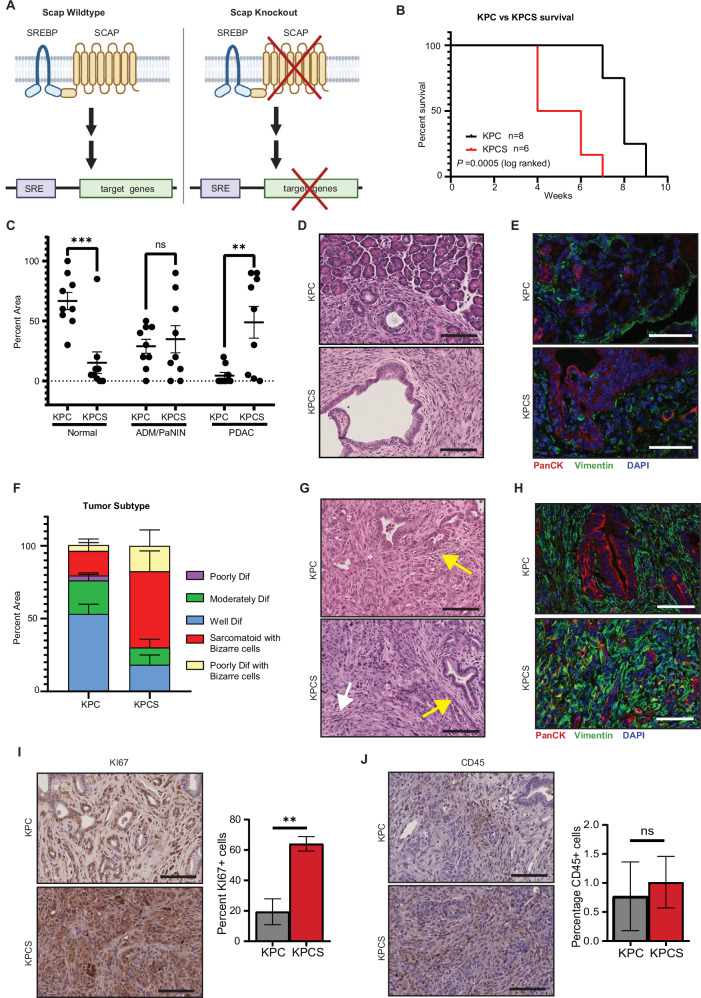
Loss of Scap accelerates and promotes loss of differentiation of KRAS/TP53 pancreatic tumors. **A** Graphical description of Scap knockout model. Created in BioRender. Lilly, A. (2026) https://BioRender.com/k5xvfzb. **B** Survival curve of KPC (*n* = 8) vs KPCS (*n* = 6) mice. **C**. Percent area of pancreata from 4-week-old KPC (*n* = 9) or KPCS (*n* = 9) mice that are normal, precancerous (ADM/PaNIN), or pancreatic cancer (PDAC). Approximately equal number males and females were analyzed per genotypes **D** Representative 20x H&E images of pancreata from 4-week-old KPC (*n* = 9) and KPCS (*n* = 9) mice. Scale bar 100 µm. **E** Representative ×40 immunofluorescence images of pancreata from 4-week-old KPC (*n* = 5) and KPCS (*n* = 5) mice stained for pan-cytokeratin (red) and vimentin (green). Scale bar 50 µm. 5 fields of view per mouse. **F** Relative area of the pancreatic tumors consisting of differentially differentiated tumor in KPC (*n* = 5) vs KPCS (*n* = 5) mice from the survival cohort. **G** Representative 20x H&E images of Scap^*Δpanc*^ and Scap^f/f^ pancreata from KPC (vs KPCS mice in the survival cohort. The yellow arrows point to representative lesions with classical PDAC. The white arrow points to a representative lesion of sarcomatoid PDAC. Scale bar 100 µm. **H** Representative 40x immunofluorescence images of pancreata from the survival study cohort (*n* = 5) and KPCS (*n* = 5) mice stained for pan-cytokeratin (red) and vimentin (green). Scale bar 50 µm. 5 fields of view per mouse. Representative 20x images for markers of (**I**) proliferation (Ki67) and **J** leukocyte infiltration (CD45) in KPC versus KPCS mice from the survival cohort, with quantitation. Scale bar 100 µm. *p ≤ 0.05; **p ≤ 0.01; ***p ≤ 0.001, ****p ≤ 0.0001, in all panels. Student’s *t* test used for all statistical analysis.

To directly test the consequence of *Scap* deletion on pancreatic tumorigenesis, we crossed *Scap*^*f/f*^ (floxed, but lacking Cre recombinase, resulting in normal *Scap* expression) and *Scap*^*wt/wt*^ mice to the well-characterized *LSL-Kras*^*G12D*^*;Trp53*^*f/f*^*;Pdx1-Cre* model of pancreatic ductal adenocarcinoma (PDAC) [8], generating KPC versus KPCS mice (Fig. 1A, Supplementary Fig S1A). The *Pdx1* promoter used to activate Cre in this model becomes active at embryonic day 8.5–9.0 in the definitive endoderm for the pancreatic bud, precursor to exocrine, endocrine, and ductal cell populations [25]. In KPC mice, signs of ill health mandating euthanasia typically occur at ~8-10 weeks of age [8]. In this study, similar results were obtained, with overall survival (OS) at ~8–9 weeks (Fig. 1A). Strikingly, in the KPCS genotype, disease progression was accelerated, with OS reduced to ~4–6 weeks (*p* = 0.0005); all mice with this genotype died of aggressive pancreatic tumors (Fig. 1B). Interestingly, we also observed a correlation between low SCAP expression and reduced survival in human PDAC, based on KM plots analysis of public data [26] (Supplementary Fig S1B).

Comparison of the pancreata of 4-week-old KPC and KPCS mice (Fig. 1C, D and Supplementary Fig S1C) revealed extensive areas of tumor tissue mass in KPCS mice, with almost no normal tissue identified, in contrast to little detected formation of tumors in KPC mice. Based on quantification of the overall cohort, 34.8% of the observed pancreatic area in KPCS mice was composed of tissue with acinar-to-ductal metaplasia (ADM) or pancreatic intraepithelial neoplasias (PanINs), and 48.9% overt PDAC (compared to 28.9% ADM/PanIN and 4.4% PDAC for KPC mice) (Fig C, D, Supplementary Fig S1C). At 4 weeks, immunofluorescence staining of KPCS pancreata revealed a large increase in vimentin-positive tissue (Fig. 1E and Supplementary Fig S1D).

Similar histopathological assessment performed on KPC and KPCS from the survival study indicated that KPCS tumors were largely sarcomatoid or poorly differentiated compared to the well to moderately differentiated KPC tumors (Fig. 1F, G and Supplementary Fig S1E, F). Additionally, a number of KPCS tumors contained bizarre cells (enlarged, often multinucleated cells associated with sarcomatoid carcinomas [27, 28]). Three of the six KPCS mice in the survival study also had areas of cystic tissue (Supplementary Fig S1G); this was not observed in KPC mice. In these specimens, vimentin staining of mesenchymal tissue was much higher in the KPCS tissues (Fig. 1H). KPCS tumors also stained more strongly for Ki67, indicating increased proliferation (Fig. 1I). In contrast, we did not observe a significant difference in staining pattern of the leukocyte marker CD45 between KPC and KPCS pancreatic tumors (Fig. 1J).

### Single nuclei transcriptomic analysis of KPC versus KPCS tumors

**Fig. 2:**
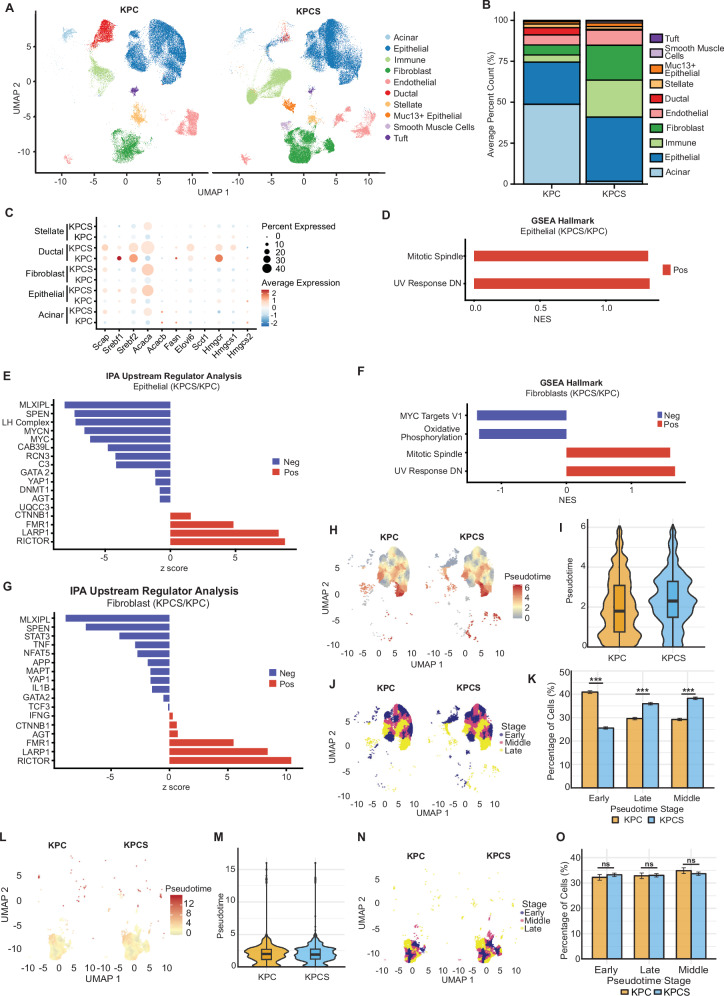
Single nuclei sequencing of KPCS and KPC pancreatic tumors. **A** UMAP of annotated clusters in 4-week-old KPCS (*n* = 2) and 7-week-old KPC (*n* = 2) single nuclei-RNA seq pancreatic samples. **B** Stacked bar graph of the average percentage of cells in each cluster per genotype/time point. **C** Dot plot showing average expression of *Scap*, *Srebf1/2*, and their downstream regulated genes in indicated cell clusters. **D** GSEA analysis of upregulated and downregulated Hallmark genesets in the 4-week-old KPCS fibroblast cluster compared to 7-week-old KPC, using fgsea. **E** IPA analysis predicting upregulated and downregulated signaling pathways responsible for altered gene expression in the 4-week-old KPCS fibroblast cluster compared to 7-week-old KPC. **F** A UMAP projection of KPC (left) and KPCS (right) epithelial cells. Point color – from blue (early pseudotime, most epithelial-like, expressing genes *Cdh1*, *Epcam*, *Krt8*, *Krt18*) through yellow (EMT) to red (late pseudotime, most fibroblastic, expressing genes *Vim*, *Fn1*, *Snai1*, *Twist1*)—reflecting Monocle 3-inferred differentiation progression, showing KPC and KPCS epithelial cells have similar patterns of differentiation. **G** A violin plot showing the quantification of the pseudotime distribution of KPC and KPCS clusters. **H** A UMAP projection of pseudotime values split into three equally populated bins (Early, Middle, and Late) using quantile-based tertile cuts. **I** GSEA analysis of upregulated and downregulated Hallmark genesets in the 4-week-old KPCS compared to 7-week-old KPC epithelial cluster, using fgsea. **J** IPA analysis predicting upregulated and downregulated signaling pathways responsible for altered gene expression in the 4-week-old KPCS compared to 7-week-old KPC epithelial cluster. **K** Bar chart of percentage of cells in KPC and KPCS samples by pseudotime stage. **L**. A UMAP projection of KPC (left) and KPCS (right) fibroblasts. Point color—from blue (early pseudotime, most myCAF-like, expressing genes *Vim*, *Des*, *Acta2*) through yellow (intermediate) to red (late pseudotime, most iCAF-like, expressing genes *Pdgfra*, *Cxcl12*, *Il6*)—reflects Monocle 3-inferred differentiation progression, showing an increase of KPCS fibroblasts expressing an iCAF phenotype compared to KPC. **M** A violin plot showing the quantification of the pseudotime distribution of KPC and KPCS clusters. **N** A UMAP projection of pseudotime values split into three equally populated bins (Early, Middle, and Late) using quantile-based tertile cuts. **O** Bar chart of percentage of cells in KPC and KPCS samples by pseudotime stage. **p ≤ 0.01, ***p ≤ 0.001, in all panels. A Chi-square test was used for quantification of percentage of cells by pseudotime stage.

To gain insight into underlying changes in cellularity and signaling induced by loss of SCAP, KPC and KPCS pancreata were collected at times corresponding to significant tumor burden (4 weeks for KPCS tumors, and 7 weeks for KPC tumors) and used for isolation of single nuclei (Fig. 2, Supplementary Fig S2A–D). For comparison, the pancreas from a 4-week-old KPC mouse was also collected for single nuclei; however, at 4 weeks of age, the sample consisted primarily of acinar cells, with no emergence of a transformed population at this time point (Supplementary Fig S2E). Because of this significant difference in cellularity between age matched KPC and KPCS pancreata, we did not continue downstream analysis involving the 4-week-old KPC mouse data. A gross comparison of cellularity following single nuclei transcriptomic analysis (Fig. 2A, B) indicated a markedly different profile between the two genotypes. Although possessing a substantial burden of tumor (epithelial) cells, KPC tumor-bearing pancreata retained extensive numbers of acinar cells, with limited populations of immune cells or fibroblasts. In contrast, the KPCS pancreata were predominantly composed of epithelial (tumor) cells, with substantial enlargement of the fibroblast and immune compartments; almost no acinar cells were detected. When comparing the subtypes of immune populations between KPC and KPCS samples, KPC samples predominantly contained macrophages, with a very minor population of dendritic cells; results similar to those previously described for this genotype in another study [29] (Supplementary Fig S2F–H). Conversely KPCS samples contained a much more diverse population, with macrophages accompanied by an expanded pool of dendritic cells and abundant lymphocytes, including both T and B cells.

Examination of the expression of SCAP/SREP and other lipid-relevant transcripts in these key populations (Fig. 2C) indicated elevated expression of *Srebf2* in the KPCS setting, and a striking elevation of *Acaca* (ACC1), a key dependency in PDAC characterized by KRAS pathway activation [30]. Gene set enrichment analysis (GSEA) and Ingenuity Pathway Analysis (IPA, [31]) were performed on differentially expressed genes (DEGs) separately on the epithelial/tumor (Fig. 2D, E), and fibroblast (Fig. 2F, G) compartments. GSEA analysis identified significant upregulation of signatures of mitotic spindle and UV response in both KPCS epithelial cells and fibroblasts, suggestive of rapidly proliferating cells, and less pronounced reduction in MYC transcriptional targets and oxidative phosphorylation in KPCS fibroblasts (Supp Table S3). Intriguingly, IPA analysis identified many common elements in the signatures of KPCS epithelial cells and fibroblasts, in comparison to matching KPC populations (Supplementary Table S4). The KPCS populations strongly downregulated MLXIPL/ChREBP and SPEN, and upregulated LARP1, and RICTOR. MLKIPL is a glucose-regulated MYC-superfamily member that activates an overlapping series of lipogenic targets with SREBPs [32]; SPEN, a transcriptional co-repressor that has been identified as a tumor suppressor in some cancers [33]. LARP1 [34] and RICTOR [35] both function as positive regulators of mTOR signaling, associated with rapid cellular growth.

Although there was an increase in sarcomatoid tumors in KPCS mice, pseudotime analysis indicated few differences in differentiation trajectory between KPC and KPCS, KPCS epithelial clusters have undergone more EMT than KPC epithelial cells (Fig. 2H–K). We also performed pseudotime characterization of the cancer associated fibroblasts (CAFs) comparing markers indicative of functional myofibroblastic CAFs (*Vim*, *Des*, *Acta2*) and/or inflammatory CAFs (*Pdgfra*, *Cxcl12*, *Il6*) within the fibroblast clusters. This analysis revealed little difference between CAF functionality in KPCS tumors compared to KPC tumors (Fig. 2K–O).

### Deletion of Scap in the pancreatic primordia disrupts pancreatic development

**Fig. 3:**
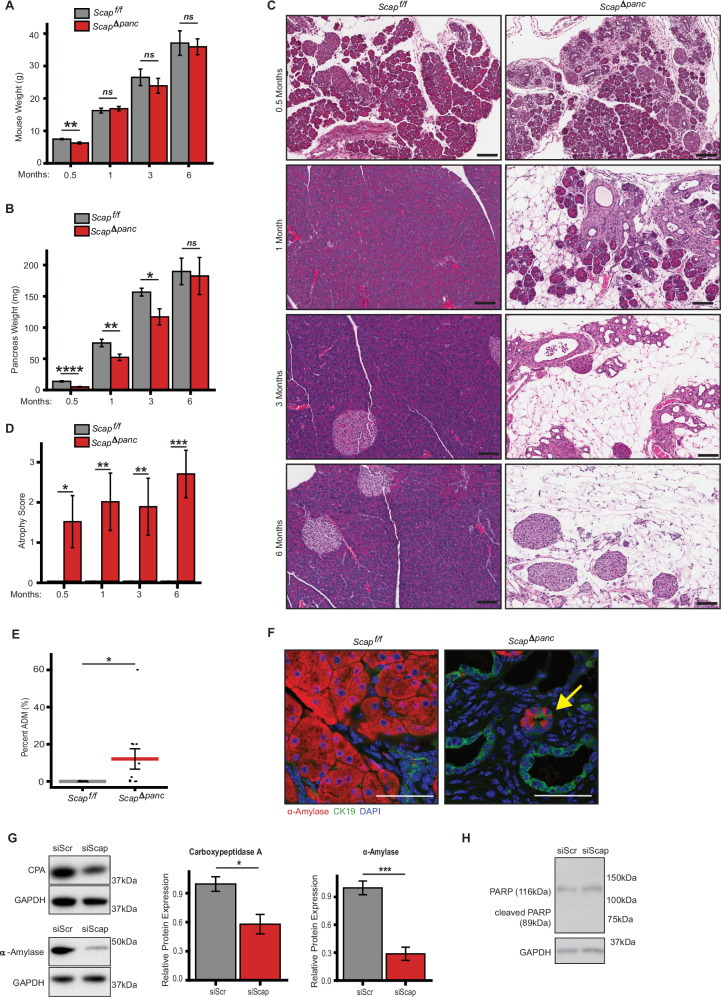
Altered pancreatic growth and cellularity in Scap^*Δpanc*^ mice. **A** Weight of *Scap*^*Δpanc*^ and *Scap*^*f/f*^ mice ± SEM at ages indicated. Analysis is based on *n* = 10 mice per genotype per age group, equal numbers male and female. **B** Weight of *Scap*^*Δpanc*^ and *Scap*^*f/f*^ pancreata ± SEM from from mice in (**A**). **C** Representative 20x H&E images of Scap^Δpanc^ and *Scap*^*f/f*^ pancreata described in (**B**). Scale bar 100 µm. **D** Pancreatic atrophy ± SEM at ages indicated. **E** Quantitation of acinar-to-ductal metaplasia (ADM) ± SEM in 2-week-old H&E stained *Scap*^*Δpanc*^ and *Scap*^*f/f*^ pancreata. **F** Representative 40x image of ADM in 1-month-old *Scap*^*Δpanc*^ pancreata compared to *Scap*^*f/f*^. Arrows indicate cells expressing both acinar (a-amylase, red)) and ductal (CK19, green)) markers. DAPI, blue. Scale bar 50 µm. 5 fields of view per mouse. **G** Left, Representative Western blot, and right, related quantitation of carboxypeptidase A (CPA) and α-amylase in AR42J cells treated with 50 nM siRNA targeting Scap or a scrambled control for 72 h (*n* = 7 independent experiments). **H** Representative Western blot of PARP in AR42J cells treated with 50 nM siRNA targeting Scap or a scrambled control for 72 h (*n* = 7 independent experiments). *p ≤ 0.05; **p ≤ 0.01; ***p ≤ 0.001, ****p ≤ 0.0001, in all panels. Student’s *t* test used for all statistical analysis.

To gain insight into the very distinct phenotypes of KPC versus KPCS tumors, we analyzed the consequences of Scap loss in the absence of PDAC oncogenic driver lesions, crossing *Scap*^*f/f*^ mice to *Pdx1-Cre* mice to create *Pdx1-Cre;Scap*^*f/f*^ mice (subsequently designated as *Scap*^*Δpanc*^*)* (Supplementary Fig S3A). *Scap*^*Δpanc*^ mice are born at normal Mendelian ratios (Supplementary Table S5). Although of lower weight than control mice at 2 weeks of age, this difference became insignificant by 1 month of age (Fig. 3A). In young mice (2–4 weeks), *Scap*^*Δpanc*^ pancreata were significantly smaller than those of control mice, although this difference also became less significant as mice age and was not observed in 6-month-old mice (Fig. 3B). In some *Scap*^*Δpanc*^ animals, pancreatic cysts were observed (Supplementary Fig S3B).

In contrast to these limited quantitative differences, histopathological analyses indicated clear differences in pancreatic development already significant in two-week-old mice, and magnified in older mice (Fig. 3C, D, and S3C, D). Severe pancreatic atrophy was observed in *Scap*^*Δpanc*^ mice, characterized by an extensive loss of acinar tissue. By 1 month of age, the exocrine compartment was largely replaced by mature adipose tissue, leaving only residual ductal structures and scattered islets of Langerhans. In several areas, ductal dilation was noted, with some ducts appearing ectatic or irregular in shape. The remaining pancreatic architecture was markedly disrupted, with minimal to no identifiable acinar cell remnants. Despite the extensive atrophy, the endocrine component (islets) appeared relatively preserved, although in some cases slightly reduced or displaced within the adipose-rich tissue. These findings suggest chronic and advanced degenerative changes, potentially resulting from long-standing injury, metabolic stress, genetic modification, or other underpinnings in the experimental model.

Comparison of the rates of proliferation and apoptosis in the pancreata of 2 week old mice (Supplementary Fig S3E, F) indicated almost no detectable apoptosis (based on caspase 3 cleavage), and no significant proliferation (Ki67) differences between the *Scap*^*f/f*^ and *Scap*^*Δpanc*^ genotypes. Rather, suggestive of a differentiation defect, numerous areas of ADM were observed in *Scap*^*Δpanc*^ pancreata (Fig. 3E, F), as immunofluorescence analysis with markers for acinar cells (α-amylase) or ductal cells (cytokeratin 19, CK19) confirmed reduced areas containing α-amylase+ acinar cells, increased areas with CK19+ ductal cells, and appearance of structures staining for both acinar and ductal markers. To determine if the observed loss of acinar cells represented a cell-intrinsic effect of SCAP loss, we knocked down *SCAP* using siRNA in the rat acinar cell line AR42J. Knockdown of *SCAP* (Fig S3G) reduced expression of the acinar markers carboxypeptidase A (CPA) and α-amylase (Fig. 3G), but did not induce apoptosis as indicated by the lack of cleaved PARP (Fig. 3H). Together, these data suggested loss of SCAP predisposed acinar cells to ADM.

### *Scap*^*Δpanc*^ mice develop features of chronic pancreatitis

**Fig. 4:**
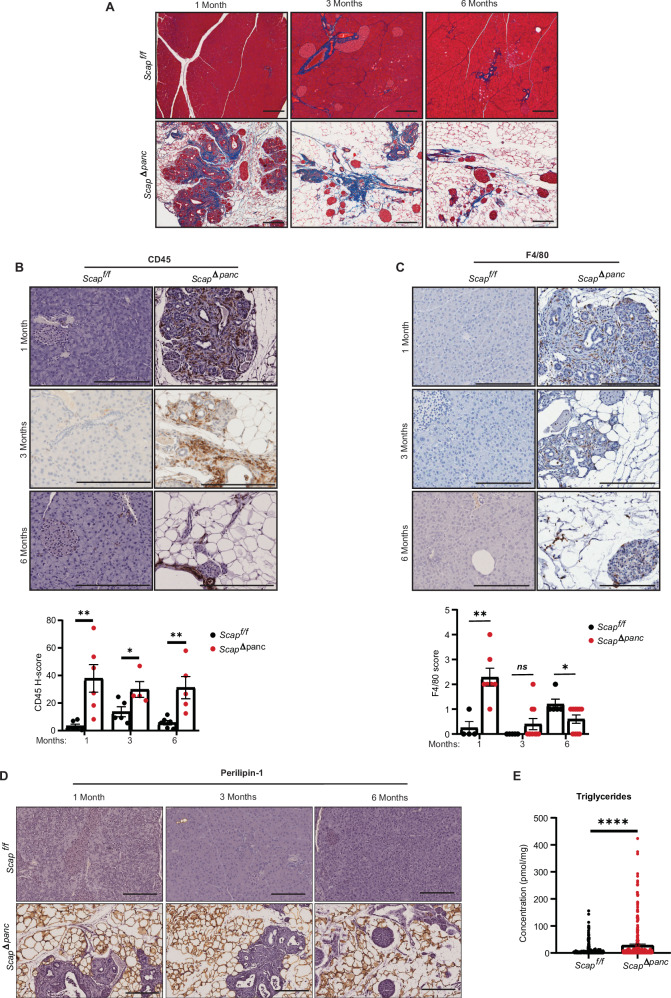
Loss of pancreatic Scap promotes fibrosis, immune infiltration, and fatty replacement. **A** Representative Masson’s trichrome staining in *Scap*^*Δpanc*^ and *Scap*^*f/f*^ pancreata from mice at ages indicated. Equal numbers of male and female mice were analyzed per genotype. Representative 20x images (**B**) of staining for markers for leukocytes (CD45, **B**) and macrophages (F4/80, **C**) in *Scap*^*Δpanc*^ and *Scap*^*f/f*^ pancreata from 1 month old, 3 months old, and 6 months old mice, and quantification of these results, ± SEM. Scale bar 100 µm. **D** Representative 20x perilipin-1 immunohistochemistry staining of *Scap*^*Δpanc*^ and *Scap*^*f/f*^ pancreata from mice at ages indicated. Analysis based on 5–6 mice per genotype, equal number males and females. Scale bar 100 µm. **E** Mean concentration of triglycerides ± SEM (pmol/mg) in *Scap*^*Δpanc*^ pancreata compared to *Scap*^*f/f*^ pancreata in 2-week-old mice. Analysis of 10 samples per genotype, equal numbers of males and females. Data has been normalized to known, spiked-in lipid controls, allowing the results to be converted to pmol of lipid/mg tissue. See also Supplementary Table S6. *p ≤ 0.05; **p ≤ 0.01; ****p ≤ 0.0001, in all panels. Student’s *t* test used for all statistical analysis.

The striking loss of acinar cells observed in mice is a typical feature of chronic pancreatitis, a pathological state associated with fibrosis, immune cell infiltration, and accumulation of adipocytes [36, 37]. Based on staining with Masson’s trichrome, we observed a significant, progressive accumulation of fibrous collagen in pancreatic tissue from *Scap*^*Δpanc*^ mice, versus little accumulation in controls, with this particularly notable in 1 month old mice (Fig. 4A). Similar results were obtained based on immunohistochemical staining with vimentin (Supplementary Fig S4A). We also observed extensive infiltration of CD45+ lymphocytes and F4/80+ macrophages into pancreatic tissue of 1 month old *Scap*^*Δpanc*^ mice (Fig. 4B, C). Accumulation of CD45+ lymphocytes was maintained 3-month-old and 6-month-old *Scap*^*Δpanc*^ mice; levels of F4/80+ macrophages were attenuated as *Scap*^*Δpanc*^ mice age (Fig. 4C).

The fatty tissue within the *Scap*^*Δpanc*^ pancreas stained intensely for perilipins-1 and -2 (Fig. 4D, Supplementary Fig S4B), proteins required for lipid droplet formation that are typically found in adipocytes, and elevated in MASLD [38]. Lipidomic analysis of *Scap*^*Δpanc*^ versus *Scap*^*f/f*^ pancreatic tissue at 2 weeks of age (Fig. 4E, Supplementary Fig S4C, Supplementary Table S6) demonstrated significant increase in triglycerides, a storage form of excess lipids often observed in pancreatitis [39], in the *Scap*^*Δpanc*^ cohort compared to the *Scap*^*f/f*^ samples. Taken in sum, these data indicated that constitutive absence of SCAP in pancreatic primordia triggered rapid onset of chronic pancreatitis.

### Lineage tracing indicates mesenchymal origin of adipocytes

**Fig. 5:**
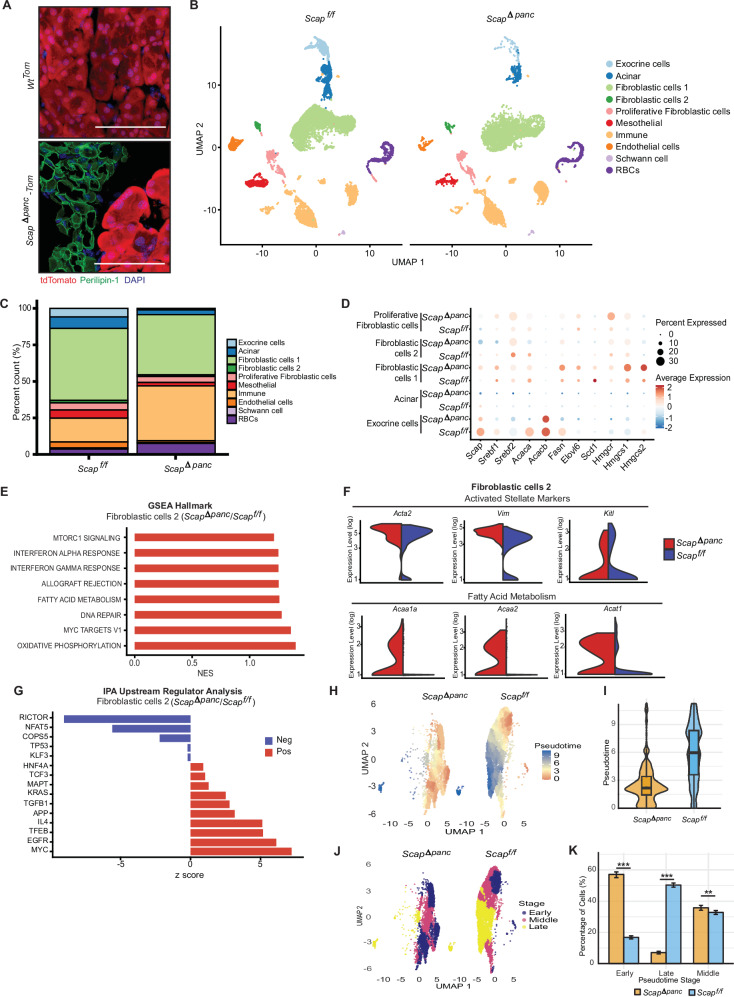
Altered cellularity and signaling in *Scap*^*Δpanc*^ and *Scap*^*f/f*^ pancreata. **A** Representative 40x images of immunofluorescence staining for Perilipin-1 (green) in *Scap*^*Δpanc*^*-Tom* (*n* = 5) and *Wt*^*Tom*^ (*n* = 4) pancreata from 1-month-old mice. Scale bar 50 µm. **B** UMAP of annotated clusters in 0.5-month-old *Scap*^*Δpanc*^ and *Scap*^*f/f*^ samples. **C** Stacked bar graph of the percentage of cells in each cluster per sample. **D** Dot plot showing average expression of *Scap*, *Srebf1/2*, and their downstream regulated genes in selected clusters, comparing *Scap*^*Δpanc*^ and *Scap*^*f/f*^ samples. **E** GSEA analysis of upregulated and downregulated Hallmark genesets in the *Scap*^*Δpanc*^ fibroblastic cell 2 cluster compared to *Scap*^*f/f*^. **F** Violin plots of gene expression associated with fibroblastic cell activation in the fibroblastic cell 2 cluster, comparing *Scap*^*Δpanc*^ and *Scap*^*f/f*^ samples. **G** Ingenuity pathway analysis (IPA) upstream regulator analysis of predicted upregulated and downregulated signaling pathways in the *Scap*^*Δpanc*^ fibroblastic cell 2 cluster compared to *Scap*^*f/f*^. **H** A UMAP projection of *Scap*^*Δpanc*^ (left) and *Scap*^*f/f*^ (right) pancreatic fibroblastic cells 1 and fibroblastic 2. Point color–from blue (early pseudotime, least differentiated, expressing genes *Vim*, *Cd44*, *Vcam1*, *Pdgfra*) through yellow (intermediate) to red (late pseudotime, most differentiated, expressing genes *Col1a1*, *Fn1*, *Acta2*, *Col6a1*)—reflects Monocle 3-inferred differentiation progression, showing that *Scap*^*f/f*^ cells advance to a later state while *Scap*^*Δpanc*^ cells remain in earlier differentiation stages. **I** A violin plot showing the quantification of the pseudotime distribution of *Scap*^*Δpanc*^ and *Scap*^*f/f*^ clusters. **J** A UMAP projection of pseudotime values split into three equally populated bins (Early, Middle, and Late) using quantile-based tertile cuts. **K** Bar chart of percentage of cells in *Scap*^*Δpanc*^ and *Scap*^*f/f*^ samples by pseudotime stage. **p ≤ 0.01, ***p ≤ 0.001, in all panels. Chi-square test used for quantification of percentage of cells by pseudotime stage.

The observations of acinar loss and replacement with adipose tissue accompanied by higher levels of storage lipids were surprising, as loss of SCAP-SREBP signaling would be expected to decrease rather than increase lipid production. To gain insight into the changes in cellularity, we crossed *Scap*^*f/f*^ versus *Scap*^*Δpanc*^ mice to *Ai9* mice, which strongly express the fluorescent protein tdTomato following Cre-dependent recombination [12]. In *Pdx1-CreAi9* mice (designated *wt*^*Tom*^) mice, the bulk of pancreatic tissue, including acinar, ductal, and endocrine tissue is uniformly tdTomato + . Notably, in *Pdx1-Cre;Scap*^*f/f*^*;Ai9* mice (designated *Scap*^*Δpanc*^*-Tom*), while the residual acinar, ductal, and endocrine tissue is tdTomato + , the adipose cells are not (Fig. 5A). Of pancreas-resident cell types, the *Pdx1* promoter is only inactive in cells of mesenchymal origin (e.g. fibroblasts, stellate cells, and adipocytes) and immune cells [40–42]. These results suggested that in mice with SCAP deficiency in exocrine and endocrine tissue, there is compensatory expansion of mesenchymal cells that subsequently differentiate into adipocytes.

### Single cell RNA sequencing identifies early reciprocal signaling changes in exocrine versus mesenchymal cell populations

To test this idea, and evaluate contributing mechanisms, we performed single cell sequencing on 2-week-old *Scap*^*Δpanc*^ and *Scap*^*f/f*^ mice. At two weeks of age, a time when relatively limited morphological differences were apparent, the overall cellular composition of the pancreas was similar between the two genotypes (Fig. 5B, Supplementary Fig S5A–C). However, a loss of acinar cell populations was already evident, as were losses in overall exocrine cells (representing immature acinar cells, ductal cells, and exocrine precursor cells) (Fig. 5C). In contrast, levels of immune cells were elevated in the *Scap*^*Δpanc*^ sample. When investigating the immune populations, the *Scap*^*Δpanc*^ sample has elevated levels of B cells and lymphocytes compared to *Scap*^*f/f*^(Supplementary Fig S5D–G). Interestingly, B cell accumulation has been linked to pancreatic inflammation [43] and has previously been reported to inhibit pancreatic epithelial regeneration in the context of chronic pancreatitis [44].

We compared the expression of genes relevant to SCAP signaling in cells of distinct lineages in *Scap*^*Δpanc*^ and *Scap*^*f/f*^ pancreata (Fig. 5D, Supplementary Fig. S5B, C, Supplementary Table S7). As expected, levels of *SCAP* itself were greatly reduced or undetectable in acinar and exocrine cells from *Scap*^*Δpanc*^ mice (Fig. 5D, Supplementary Fig S5B), whereas little difference was seen in the fibroblastic 1 cluster (with an expression profile most similar to local resident fibroblasts, defined based on intermediate expression of collagen genes, *Fn1, Pdgfra, Fbn1, Fndc1*, and *S100a11*) or the fibroblastic 2 cluster (with an expression profile similar to stellate cells: *Acta2*^*+*^*Pdgfra*^*low*^). Similar results were obtained for *Srebf1* expression in all analyzed cell populations (Fig. 5D, Supplementary Fig S5B). For SREBP2, while significant reduction in expression was observed in acinar and exocrine clusters from *Scap*^*Δpanc*^ pancreata, the degree of reduction was much less in ductal and endocrine cells than was observed for *Srebf1*. Notably, *Srebf2* expression was significantly elevated in *Scap*^*Δpanc*^ fibroblastic 1 and fibroblastic 2 clusters. SREBPs direct the transcription of genes including *Fasn, Acaca/b, Scd1, Elovl6, Hmgcr*, and *Hmgcr1/2*, required for lipid production and uptake. For these genes, we observed a pattern similar to *Srebf2*, with reduced expression in acinar and exocrine cells. In contrast, expression of these genes was elevated in *Scap*^*Δpanc*^ fibroblastic cells (Fig. 5D).

We performed GSEA on DEGs in discrete *Scap*^*Δpanc*^ versus *Scap*^*f/f*^ cell clusters. While no statistically significant changes were identified in fibroblastic 1 cells, *Scap*^*Δpanc*^ fibroblastic 2 cells had elevated expression of Hallmark gene sets for fatty acid metabolism (including *Acaa1, Acaa2*, and *Acat1)*, as well as for mTORC1 and MYC, DNA repair, IFNγ, and oxidative phosphorylation, associated with proliferation and inflammation (Fig. 5E, Supplementary Table S3). Notably, increased fatty acid metabolism and fatty acid oxidation have been previously reported to increase in activated stellate cells [45–47]. Additional fibroblastic markers such as *Acta2* and *Fn1* were also elevated in *Scap*^*Δpanc*^ cells compared to *Scap*^*f/f*^ (Fig. 5F). IPA identified a complex pattern of transcriptional changes in *Scap*^*Δpanc*^ fibroblastic 2 cells, including significant upregulation of KRAS and MYC-dependent transcription and downregulation of TP53-dependent transcription, among others (Fig. 5G). To assess if fibroblastic differentiation is affected by the loss of Scap in pancreatic exocrine and endocrine tissue, we performed pseudotime analysis on the combined fibroblastic 1 and fibroblastic 2 clusters (the combined set referred to as fibroblastic). This indicated that the *Scap*^*Δpanc*^ fibroblastic cells are restricted to an earlier pseudotime stage expressing more mesenchymal progenitor markers (*Vim, Cd44, Vcam1, Pdgfra*) while *Scap*^*f/f*^ fibroblastic cells are found primarily in the middle to late stage associated with more differentiated fibroblastic markers (*Col1a1, Fn1, Acta2, Col6a1*) (Fig. 5H, K).

**Fig. 6:**
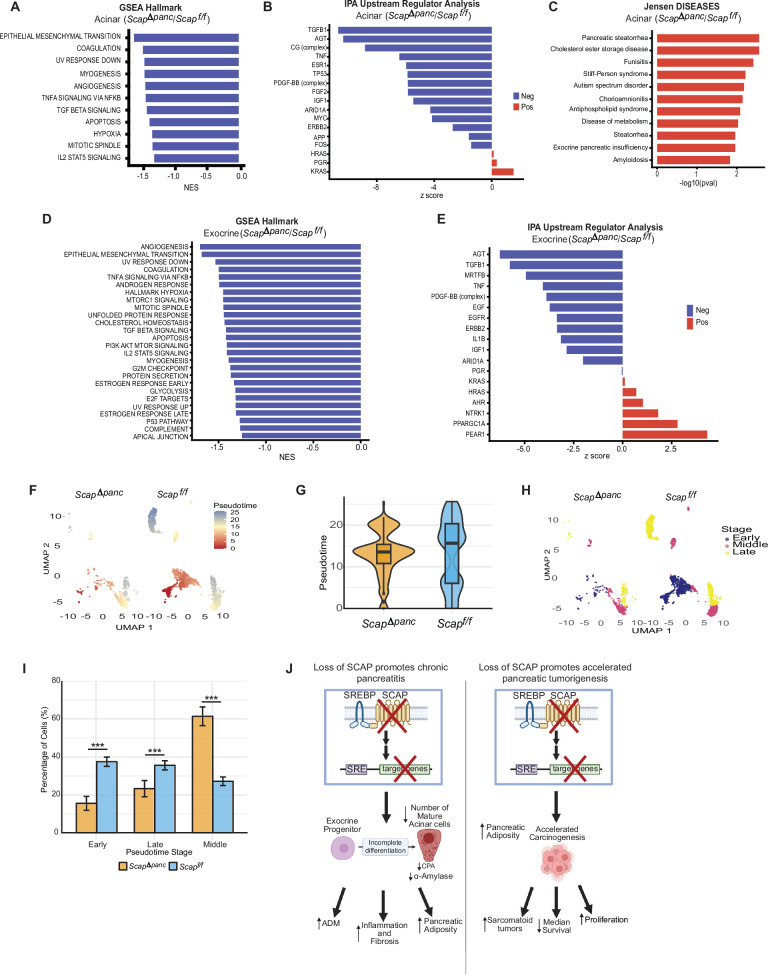
Altered signaling in *Scap*^*Δpanc*^ and *Scap*^*f/f*^ acinar and exocrine cells shows developmental impact of Scap loss. **A** GSEA analysis of upregulated and downregulated Hallmark genesets in the *Scap*^*Δpanc*^ compared to *Scap*^*f/f*^ acinar cluster. **B** IPA upstream regulator analysis of predicted upregulated and downregulated signaling pathways in the *Scap*^*Δpanc*^ acinar cluster compared to *Scap*^*f/f*^. **C** Enrichr analysis of Jensen DISEASES genesets associated with genes overexpressed in *Scap*^*Δpanc*^ versus *Scap*^*f/f*^ acinar cells. **D** GSEA analysis of upregulated and downregulated Hallmark genesets in the *Scap*^*Δpanc*^ compared to *Scap*^*f/f*^ exocrine cluster. **E** IPA analysis of predicted upregulated and downregulated signaling pathways associated with observed gene expression changes in the *Scap*^*Δpanc*^ versus *Scap*^*f/f*^ exocrine cluster. **F** A UMAP projection of *Scap*^*Δpanc*^ (left) and *Scap*^*f/f*^ (right) pancreatic exocrine and acinar cells. Point color—from blue (early pseudotime, least differentiated, expressing genes *Pdx1*, *Hnf1b*, *Sox9*, *Ptf1a*) through yellow (intermediate) to red (late pseudotime, most differentiated, expressing genes *Amy2a*, *Cpa1*, *Prss1*, *Krt19*)—reflects Monocle 3-inferred differentiation progression, showing that *Scap*^*Δpanc*^ cells are restricted to the middle differentiation stages while *Scap*^*f/f*^ cells had both earlier and later differentiation states. **G** A violin plot showing the quantification of the pseudotime distribution of *Scap*^*Δpanc*^ and *Scap*^*f/f*^ clusters. **H** A UMAP projection of pseudotime values split into three equally populated bins (Early, Middle, and Late) using quantile-based tertile cuts. **I** Bar chart of percentage of cells in *Scap*^*Δpanc*^ and *Scap*^*f/f*^ samples by pseudotime stage. **J** Graphical abstract describing how the loss of Scap impacts the pancreas. Created in BioRender. Lilly, A. (2026) https://BioRender.com/511uib5. ***p ≤ 0.001, in all panels. Chi-square test used for quantification of percentage of cells by pseudotime stage.

Given the observation of ADM and potential defect in differentiation of acinar cells in *Scap*^*Δpanc*^ pancreata, we separately analyzed signaling in acinar versus the broader exocrine compartment. In the acinar cluster, GSEA analysis indicated the significant decrease in genesets including TGFβ signaling and EMT, TNFα signaling, and angiogenesis/hypoxia, in *Scap*^*Δpanc*^ versus *Scap*^*f/f*^ samples (Fig. 6A). IPA upstream regulator analysis separately indicated the strongest downregulation of TGFβ1-dependent transcription in *Scap*^*Δpanc*^ acinar cells, also finding downregulation of TP53-, and AGT-transcription (Fig. 6B); concomitant with elevated KRAS and HRAS transcription (Fig. 6B). Intriguingly, AGT, encoding angiotensin, controls the renin-angiotensin system that regulates blood pressure and vascular function, but has also been linked to regulation of adipogenesis and cross-regulation with SREBP1 [48, 49]. Further, analysis using EnrichR [18] of significantly upregulated genes in the *Scap*^*Δpanc*^ acinar cluster (Fig. 6C) identified significant overlap with genes elevated in exocrine pancreatic insufficiency and pancreatic steatorrhea, a condition characterized by excess amounts of fat; as well as syndromes related to metabolic disfunction such as cholesterol ester storage disease and disease of metabolism (Fig. 6C). GSEA analysis of the exocrine cluster was similar to that of acinar cells, with a significant decrease in TGFβ signaling, EMT, TNFα signaling, and angiogenesis/hypoxia in *Scap*^*Δpanc*^ versus *Scap*^*f/f*^ samples (Fig. 6D). Additionally, the exocrine *Scap*^*Δpanc*^ cluster had a decrease cholesterol homeostasis, consistent with the loss of SCAP/SREBP signaling. IPA analysis also yielded results similar to the acinar cluster (Fig. 6E), with some additions; the pancreatic tumor suppressor MRTFB [50] was significantly downregulated, while PEAR1, a promoter of fibroblast activation and fibrosis [51], was strongly elevated.

Using pseudotime analysis to compare cell differentiation pathways, we investigated if the loss of SCAP impacts the trajectory of differentiation of specific pancreatic populations. We first verified the expression profiles of acinar (*Cela2a, Cpa1, Cpa2, Pnliprp1, Try4)* and ductal (*Sox9, Krt19, Muc1*) markers in *Scap*^*Δpanc*^ and *Scap*^*f/f*^ samples (Supplementary Fig S6A, B). These UMAPs show the majority of clusters express acinar markers, with a small cluster expressing ductal markers, and clusters expressing both acinar and ductal markers. The expression of *Scap*, *Srebf1/2*, and SCAP/SREBP signaling targets (*Ldlr*, *Acaca*, *Fasn*) was uniformly decreased in *Scap*^*Δpanc*^ versus *Scap*^*f/f*^ samples (Supplementary Fig S6C). Using pseudotime, we observed that *Scap*^*Δpanc*^ cells stalled at the middle pseudotime stage compared to *Scap*^*f/f*^ (Fig. 6F, G), indicating a delay in progression toward a terminally differentiated acinar states. When comparing the trajectory-based clustering between *Scap*^*f/f*^ and *Scap*^*Δpanc*^, we observed an intermediate subpopulation almost exclusively composed of *Scap*^*Δpanc*^ cells at mid-pseudotime (*p* < 0.001; Fig. 6H, I), consistent with incomplete acinar differentiation.

To further investigate how SCAP loss stalls acinar cell terminal differentiation, we looked at the expression of acinar differentiation markers, genes associated with pancreatic steatosis, and genes associated with pancreatic cancer (Supplementary Fig S7). Although the very small numbers of *Scap*^*Δpanc*^ acinar cells at early stages of differentiation limited conclusions, expression of two genes essential for acinar cell differentiation and maintenance, *PTF1a* and *NR5A22*, appeared moderately reduced in *Scap*^*Δpanc*^ cells compared to *Scap*^*f/f*^ cells, as was expression of the tumor suppressor *TRP53*. Finally, a similar phenotype of global acinar replacement by adipose tissue has previously been observed in other studies involving induced loss or gain of genes in the pancreatic exocrine/endocrine primordia, as reviewed in [37]. Studies using the *Pdx1* promoter to eliminate genes including cMYC [52], IKKa [53], JAG1 [54], KIF3A [55], or PROX1 [56], or a Ptf1 promoter to overexpress TGFBR1 in acinar cells [57] in each case identified progressive acinar loss and replacement with adipose cells, in some cases accompanied by ADM and fibrosis. However, in *Scap*^*Δpanc*^ cells, expression of mRNA for *MYC, JAG1* and other genes was relatively unaffected.

## Discussion

The work reported in this study supports several conclusions (model in Fig. 6J). First, in KPCS mice, the normal program of PDAC development is greatly accelerated versus that in KPC mice, and tumor differentiation trajectory is altered, including the appearance of pancreatic cysts, and more sarcomatoid/mesenchymal tumors. Second, single nuclei analysis of KPCS versus KPC tumors indicates a substantial expansion of the fibroblast and immune compartments in KPCS tumors, with both tumor and fibroblasts upregulating activators of the pro-tumoral mTOR signaling pathway, and signatures indicating rapid growth. Third, efficient differentiation and maintenance of acinar cells in vivo require an intact SCAP-SREBP signaling axis. Fourth, acinar cells are more sensitive than other exocrine and endocrine lineages to loss of SCAP-SREBP signaling. Fifth, global absence of SCAP in the pancreatic exocrine- and endocrine-producing primordia triggers reactive changes in all mesenchymal populations, leading to progressive differentiation towards adipocytes, and concomitant acquisition of features of pancreatitis, including fibrosis and inflammation. Sixth, PSC and mesenchymal cells from pancreata, including PSCs and other fibroblasts, of *Scap*^*Δpanc*^ mice display an altered transcriptional program, with upregulation of SREBP2, mTORC, and interferons, contributing to the creation of a pro-inflammatory milieu.

Some of the phenotypes in this study are reminiscent of those in a recent study by Kawamura and colleagues that was based on the observation that SREBP-mediated de novo lipogenesis are upregulated in NAFLD and hepatocellular carcinoma (HCC), causing SREBP inhibition to be proposed as a therapeutic strategy. The authors therefore investigated deletion of *Scap* as a potential way to ameliorate symptoms NAFLD, NASH and formation of liver cancer, using an *Alb-Cre;Pten*^*flox*^ model of NASH [58]. While in that study, deletion of *Scap* reduced hepatic steatosis (contrary to the results shown here), it unexpectedly revealed that *Scap* deletion promoted liver fibrosis and inflammation marked by infiltration of macrophages and CD45+ cells. SCAP deletion also accelerated formation of HCC, with tumors marked by hyperactivation of the mTOR signaling pathway. Increasing confidence, many of these findings were then validated in choline-deficient, l-amino acid–defined, high-fat-diet (CDAHFD) mice, an independent model of NASH-HCC [58]. The authors proposed a mechanism by which lipid imbalance led to ER stress, which promoted liver injury, cell death, and carcinogenesis.

In contrast to the findings in liver, the loss of SCAP in endodermally derived pancreatic cells (based on *Pdx1* promoter-driven *Scap* loss) in the present study was associated with a defect in exocrine cell differentiation, and an increase rather than decrease in mesenchymal adiposity. The data presented here indicate extensive crosstalk between exocrine and fibroblastic compartments in the early post-natal phase, with elevated SREBP-dependent signaling in fibroblastic cells, over-compensating for SCAP loss; the activated stromal phenotype of the fibroblasts in *Scap*^*Δpanc*^ pancreata is often tumor promoting [59]. In this context, it is intriguing that a number of studies identifying acinar loss/adipose replacement phenotypes also identified defects in pancreatic cilia [54, 60–63], a structure known to mediate signaling between pancreatic cancer precursors and fibroblasts in the proto-tumor microenvironment [64]. Notably, loss of SCAP elevated ADM, a precursor to formation of pancreatic intraepithelial neoplasias (PanINs) [65], both in the presence or absence of KRAS and Trp53 driver lesions.

This study aligns with a growing body of literature that describes dialog between cancer cells and adjacent fibroblasts that results in activation and reprogramming of lipid production in CAFS, with the reprogrammed CAFs then providing lipids to fuel the growth of cancer cells [66–68]. Although the reprogramming mechanism is not clear, and may vary across distinct tumor types, data in this study indicates upregulation of SREBP2 in fibroblastic cells likely contributes to the reprogramming. This may be SCAP-dependent, as SCAP would be intact in pancreatic fibroblasts; alternatively, some SCAP-independent mechanisms of SREBP activation have been described [69, 70]. One intriguing element of the phenotypes reported here are the presence of abundant adipocytes when *Scap* ablation occurs in isolation, versus no adipocytes when this occurs in parallel with triggering of driver *Kras* and *Trp53* mutations. A likely possibility is that the combined signaling downstream of combined *Scap*, *Kras*, and *Trp53* lesions changes the trajectory of acinar differentiation so that these cells no longer produce pro-adipogenic signals that act on adjacent fibroblasts. These results suggest a number of future directions of interest. For example, because the focus on SCAP this work was nominated by earlier work by Gabitova-Cornell et al., which used a *Trp53*-null KPC pancreatic cancer mouse model [8], we have used a similar experimental design, as opposed to an alternative KPC mouse model bearing a mutant form of *Trp53*. It is possible that mutation rather than loss of *Trp53* may lead to a milder cancer phenotype. It would also be of interest to investigate tumorigenesis in an inducible model (e.g. [71]), to separate potential effects affecting pancreatic precursors from those induced by loss of SCAP at later stages.

Although there has been relatively little focus on SCAP in human cancer, some reports have linked SCAP function to cancer risk, cancer presentation, and therapeutic response, and KMPlot analysis indicates that lower SCAP expression was associated with worse survival in human PDAC [26]. In earlier work, we have demonstrated that inhibition of the cholesterol signaling pathway to induce SREBPs in pancreatic cancer cells elevates expression TGFB1 [8], a potent regulator of mesenchymal function that signals through TGFBR1, causing an epithelial-to-basal lineage transition that is associated with an aggressive PDAC phenotype. This relationship was echoed in analysis of sera from human patients with PDAC, suggesting a basal phenotype with use of statins [8]. The fact that our data shows that that loss of SCAP/SREBP signaling exacerbates pancreatic cancer in the KPC mouse model, suggesting that the blockade of SREBP signaling may activate alternate pathways to promote pancreatic cancer. Some of these pathways may be cancer cell intrinsic; however, our results some involve paracellular signaling that activates fibroblasts and the immune microenvironment.

In other cancer types, overexpression of SCAP in human hepatocellular carcinoma (HCC) has ben associated with hypercholesterolemia and resistance to sorafenib, and inhibition of SCAP increased sorafenib sensitivity [72]. Elevated intracellular glucose in EGFRvIII glioblastoma promotes SCAP glycosylation, disrupting its interaction with INSIG and driving SREBP-dependent transcription; inhibition of SCAP glycosylation impaired xenograft glioblastoma growth [73]. SCAP has been linked to breast cancer susceptibility in a recent transcriptome-wide association studies (TWAS) screen for alternative polyadenylation, a process that can control translation of target mRNAs, and localization and protein interactions of the encoded protein [74]. Polymorphic variants of SCAP have been reported, and some linked to incidence of obesity (e.g. [75]). In the context of data presented here, one particularly interesting recent study has correlated very low activity of SCAP/SREBP signaling with greater severity of fibrosis in human MASLD/MASH [58].

Cumulatively, these and other reports support a modifier role for SCAP in cancer. Clearly, the study presented here differs from the prior literature in that SCAP is absent throughout pancreatic development, and SCAP function is lost selectively in exocrine/endocrine pancreatic lineages, rather than throughout the affected organ. However, together with earlier studies, this work emphasizes the importance of SCAP in conditioning cancer cell signaling, and emphasize the complexity of targeting SCAP in a heterocellular organ, with distinct cell types having discrete requirement for SCAP function, or modifying differentiation course to compensate for SCAP loss. Given the broad interest of the cancer community in therapeutic targeting of the cholesterol pathway [76, 77], or SCAP-SREBP signaling specifically [78], these are important considerations.

## Supplementary information

Supplemental Table and Figure Legends; Supplemental Figures; Supplemental Methods

Supplemental Table S1

Supplemental Table S2

Supplemental Table S3

Supplemental Table S4

Supplemental Table S5

Supplemental Table S6

Supplemental Table S7

## Data Availability

The data generated in this study are available within the article and its supplementary data files. The sequencing data generated in this study are publicly available in Gene Expression Omnibus (GEO) at GSE306231 and GSE317919.
