# Supplementary material for: Role of SCAP in regulation of pancreatic homeostasis, pancreatitis, and tumorigenesis
Source: Oncogene. 2026 Apr 15;45(21):1999–2013. doi: 10.1038/s41388-026-03784-y (PMC13190320; doi:10.1038/s41388-026-03784-y)
Supplement: Supplementary file 1 — Supplemental Table and Figure Legends; Supplemental Figures; Supplemental Methods [file 41388_2026_3784_MOESM1_ESM.docx]

**Lilly *et al*., Role of SCAP in regulation of pancreatic homeostasis, pancreatitis, and tumorigenesis**

**Supplemental Figure and Table Legends; Supplemental Figures; Supplemental Methods.**

***All Supplemental Tables are provided as accompanying Excel files*:**

**Supp Table S1.** Primer information.

**Supp Table S2.** Antibody information.

**Supp Table S3.** Data table related to GSEA analysis/Excel.

**Supp Table S4.** Data table related to IPA.

**Supp Table S5.** A Scap^Δpanc^ genotype does not induce pre-natal mortality.

**Supp Table S6.** Comparison of lipids in Scap^Δpanc^ and *Scap^f/f^* pancreata from 2-week-old mice.

**Supp Table S7**. Data table of differentially expressed genes.

**Supp Fig S1. Aggressive phenotypes of KPCS versus KPC tumors. A.** Representative PCR of genotyping KPCS mice. **B.** Survival plot generated from KM Plot showing the relationship between *SCAP* expression and overall survival of PDAC patients [1]. **C.** Additional 20x H&E images of 4-week-old KPC (n=9) and KPCS (n=9) mice in which each field is from a distinct mouse. 9 mice per genotype. Scale bar 100 μm **D.** Additional 40x immunofluorescence images (pan-cytokeratin (panCK, red), vimentin (green), DAPI (blue)) of 4-week-old KPC (n=5) and KPCS (n=5) mice. 5 fields of view observed per mouse. Scale bar 50 μm. **E.** Additional 20x H&E images of survival KPC (n=8) and KPCS (n=6) mice. Scale bar 100 μm. **F.** Additional 40x immunofluorescence images (pan-cytokeratin (panCK, red), vimentin (green), DAPI (blue)) of survival KPC (n=5) and KPCS (n=5) mice. 5 fields of view observed per mouse. Scale bar 50 μm. **G.** 2x H&E of KPC (n=8) and KPCS (n=6) tumors from survival cohort indicating cystic regions in KPCS tumors, scale bar 1 mm.

**Supp Fig S2. Profiling single nuclei RNA sequencing samples from tumor samples. A.** Violin plots (log scale) of the mitochondrial percentage, UMI and features in samples after applying QC cutoffs. **B.** UMAP of clusters from combined tumor samples. **C.** Heatmap showing expression patterns of genes used to determine cell type of each cluster. **D.** Dot plot showing average expression of *Scap*, *Srebf1/2*, and their downstream regulated genes in all clusters, comparing KPC and KPCS tumor groups. **E.** Split UMAP of clusters from tumor samples (7-week-old KPC samples and 4-week-old KPCS samples) and a 4-week-old KPC sample. **F.** Expression of *Ptprc*, which encodes CD45, in tumors (KPC 7 weeks and KPCS 4 weeks) samples. **G.** Stacked bar graph of immune clusters from tumor (7-week-old KPC samples and 4-week-old KPCS samples). **H.** Dot plot showing average expression of genes related to immune cell differentiation.

**Supp Figure S3. Consequences of SCAP deletion or depletion in pancreatic cells**. **A**. Representative 2% agarose gel of PCR genotyping results for *Scap^flox^* and *Cre*. **B.** Representative 20x images of cystic regions in *Scap^Δpanc^* 0.5- and 1-month old tissue; each image is from an independent mouse. Scale bar 100 μm. **C.** Immunohistochemical staining for insulin in islet cells of *Scap^Δpanc^* and *Scap^f/f^* pancreata from 1-month-old, 3-month-old, and 6-month-old mice. **D.** Representative images of 40x immunofluorescence staining (α-amylase (red); CK19 (green); DAPI (blue)) in *Scap^Δpanc^* and *Scap^f/f^* pancreata from 0.5-, 3, and 6-month-old mice. Quantification of α-amylase and CK19 ± SD are shown below. 5 samples per genotype, balanced male and female. **E.** Representative image of caspase 3 staining in 0.5-month-old *Scap^Δpanc^* and *Scap^f/f^* pancreata. **F.** Quantification and representative images of Ki67 staining in pancreata from 2- and 4-week-old Scap^Δpanc^ and *Scap^f/f^* mice. **G.** Quantification of qRT-PCR results in AR42J cells treated with 50nM siRNA targeting Scap or a scrambled control for 72 hours (n=4 independent experiments). ***,p  ≤ 0.001, in all panels. Student’s t-test used for all statistical analysis.

**Supp Fig S4. Fibrosis and altered lipid profile in *Scap^Δpanc^* versus *Scap^f/f^* pancreata**. **A**. Representative 40x immunofluorescence images of vimentin (green), and DAPI (blue) staining in *Scap^Δpanc^* and *Scap^f/f^* pancreata from 1-, 3-, and 6-month-old mice. **B**. Representative perilipin-2 immunohistochemistry staining of *Scap^Δpanc^* and *Scap^f/f^* pancreata from mice at ages indicated. Analysis based on 5-6 mice per genotype, ~equal number males and females. **C**. Volcano plot of differentially expressed lipids in *Scap^Δpanc^* compared to *Scap^f/f^* pancreata in 2-week-old mice. Analytes are pooled from 10 samples per genotype, equal numbers of males and females. Data has been normalized to known, spiked-in lipid controls, allowing the results to be converted to pmol of lipid/mg tissue.

**Supp Fig S5. Profiling single cell RNA sequencing samples from 0.5-month-old in *Scap^Δpanc^* and *Scap^f/f^* pancreata. A.** Violin plots (log scale) of the mitochondrial percentage, unique molecular identified (UMI) and genes (features) in *Scap^Δpanc^* and *Scap^f/f^* samples after applying quality control (QC) cutoffs. **B.** UMAP of clusters from pooled 2-week-old samples (inclusive of *Scap^Δpanc^* and *Scap^f/f^*). **C.** Heatmap showing expression patterns of genes used to determine cell type of each cluster. **D.** Feature plot of *Ptprc* expression in tumor *Scap^Δpanc^* and *Scap^f/f^* samples. **E.** Split UMAPs of immune clusters in *Scap^Δpanc^* and *Scap^f/f^* samples. **F.** Stacked bar graph of immune clusters *Scap^Δpanc^* and *Scap^f/f^* samples. **G.** Dot plot showing average expression of genes related to immune cell differentiation and lineage.

**Supp Fig S6. Profiling expression profiles of acinar and exocrine clusters from from 0.5-month-old in *Scap^Δpanc^* and *Scap^f/f^* pancreata. A.** UMAPs of mature acinar cell markers (*Cela2a, Cpa1, Cpa2, Pnliprp1, Try4*) in *Scap^Δpanc^* and *Scap^f/f^* specimens. **B.** UMAPs of ductal cell markers (*Sox9, Krt19, Muc1*) in *Scap^Δpanc^* and *Scap^f/f^* specimens. **C.** UMAPs of Scap/Srebp signaling markers in *Scap^Δpanc^* and *Scap^f/f^* specimens.

**Supp Fig S7. UMAPs of genes associated with acinar differentiation, pancreatic steatosis, and pancreatic cancer. A.** UMAPs of acinar differentiation markers (*Ptf1a*, *Rbpjl*, *Nr5a2*). **B.** UMAPs of genes whose loss is associated with pancreatic steatosis (*Myc*, *Jag1*). **C.** UMAP of *Trp53*, a gene associated with pancreatic cancer.


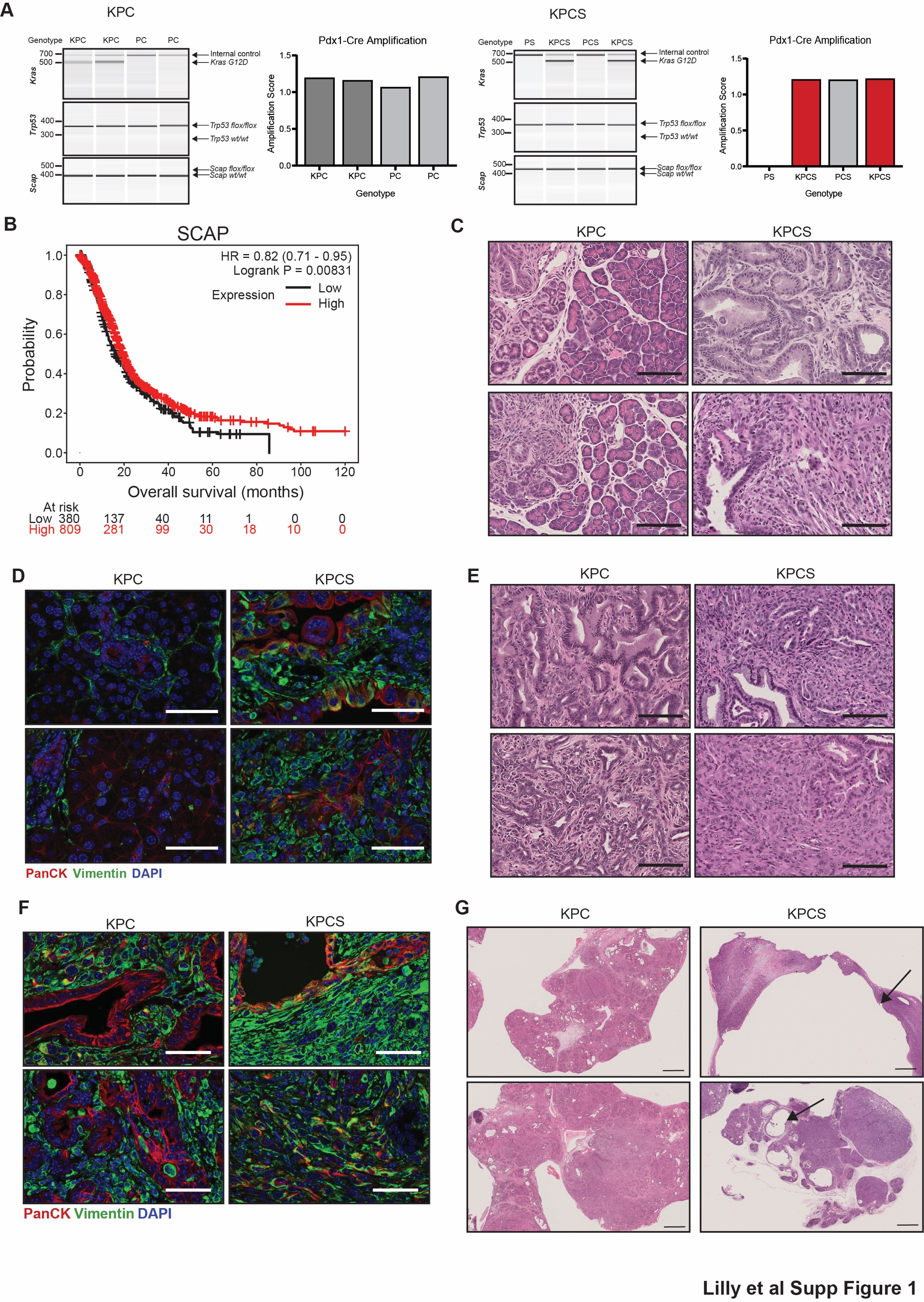

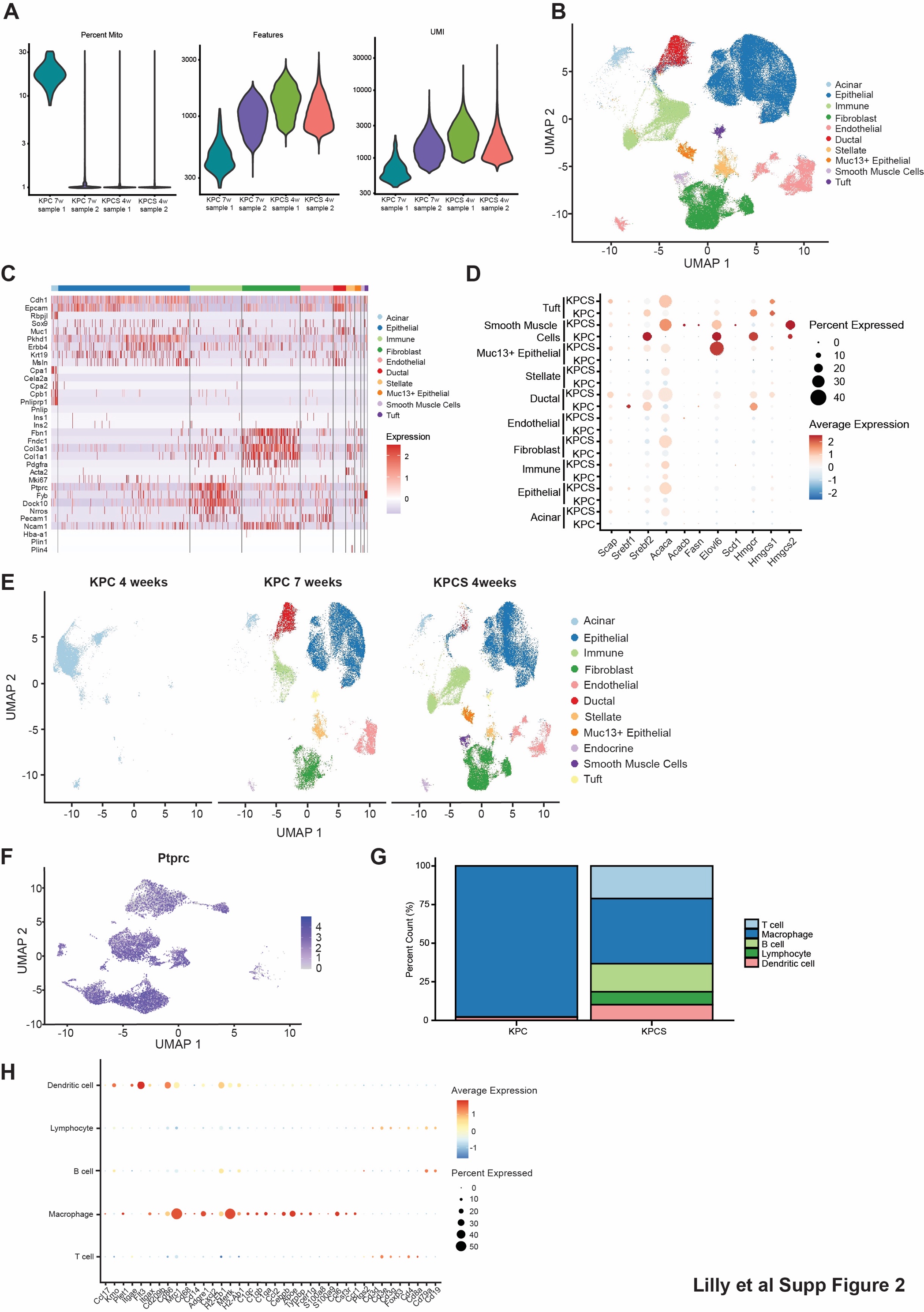


**
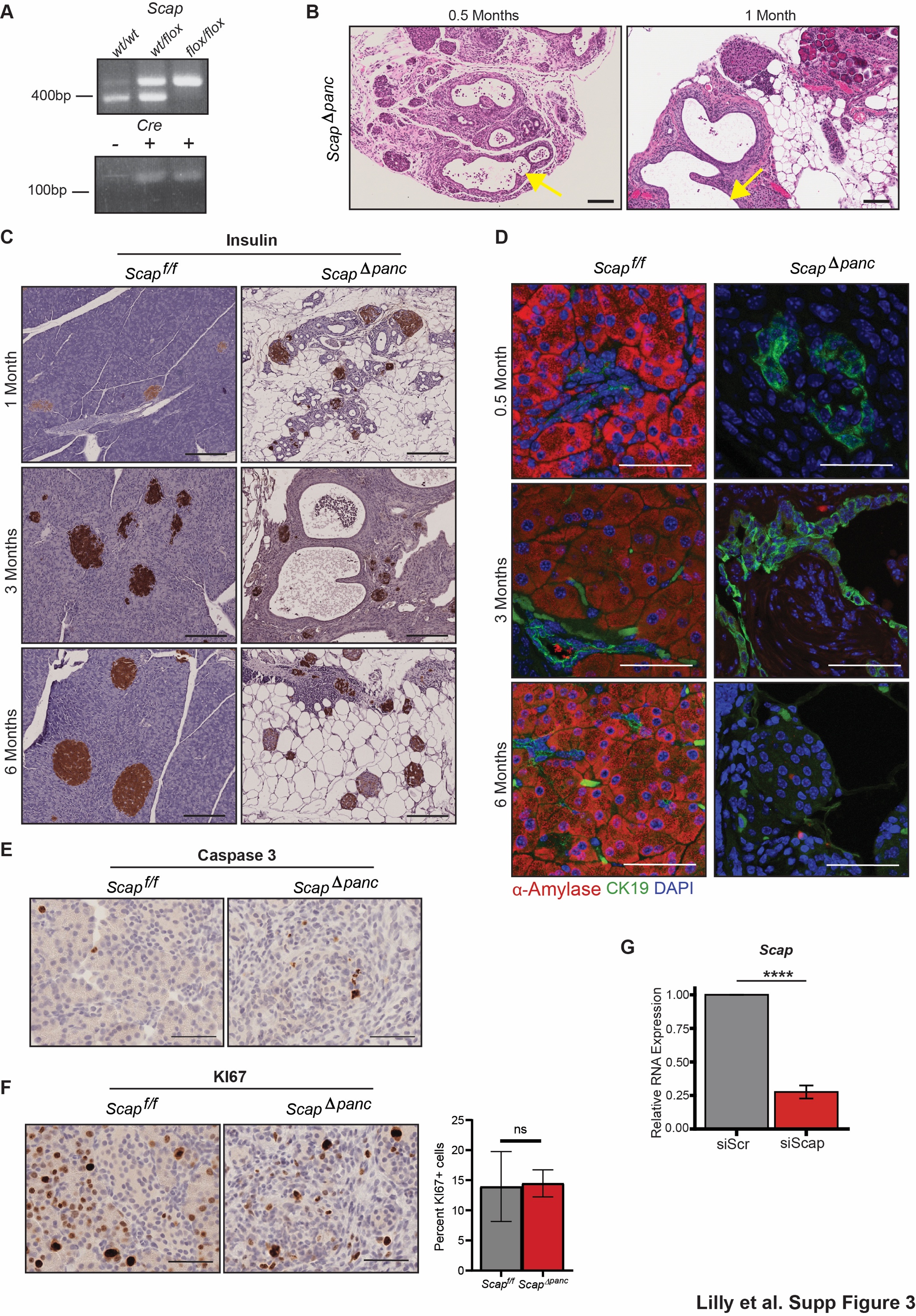
**


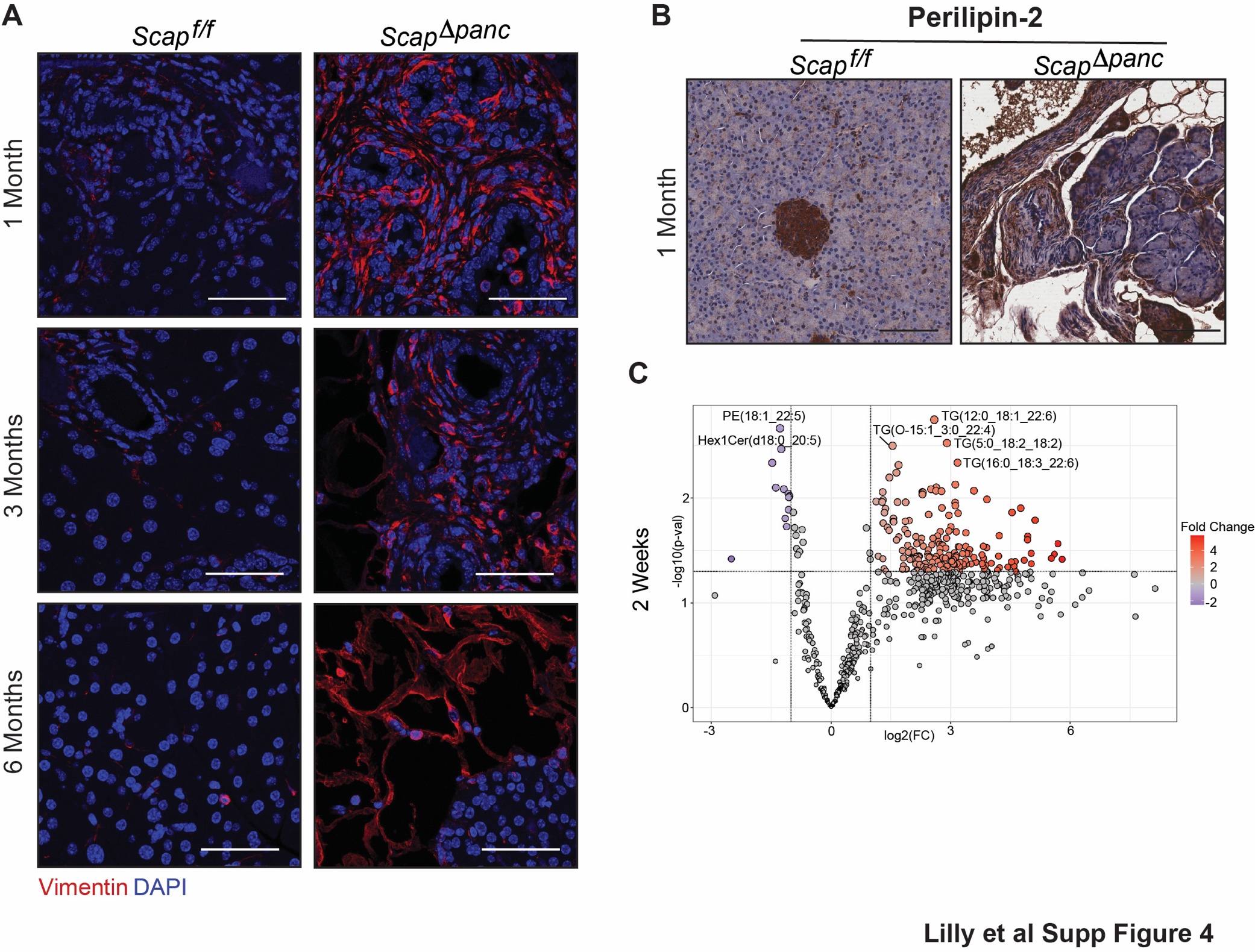


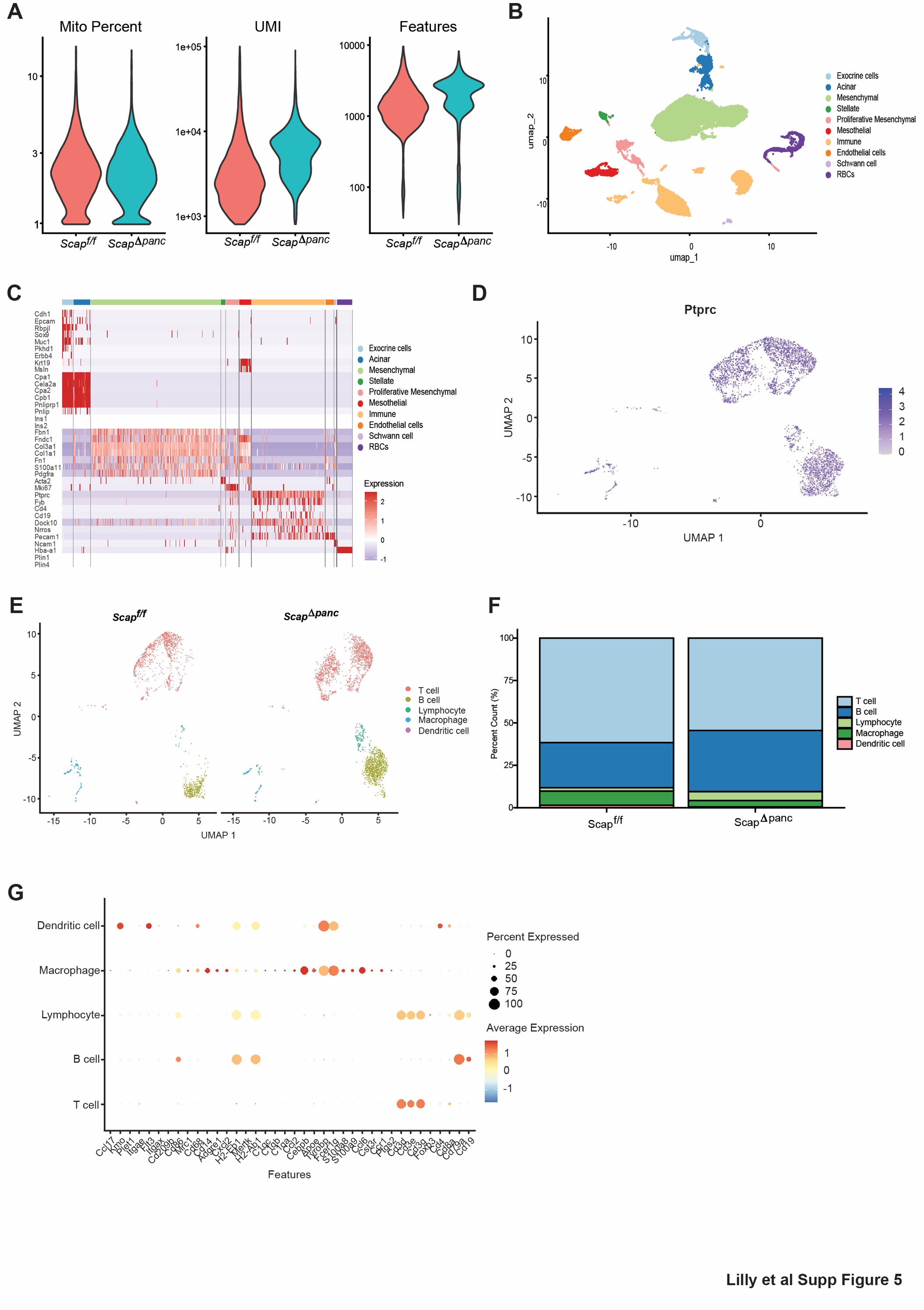


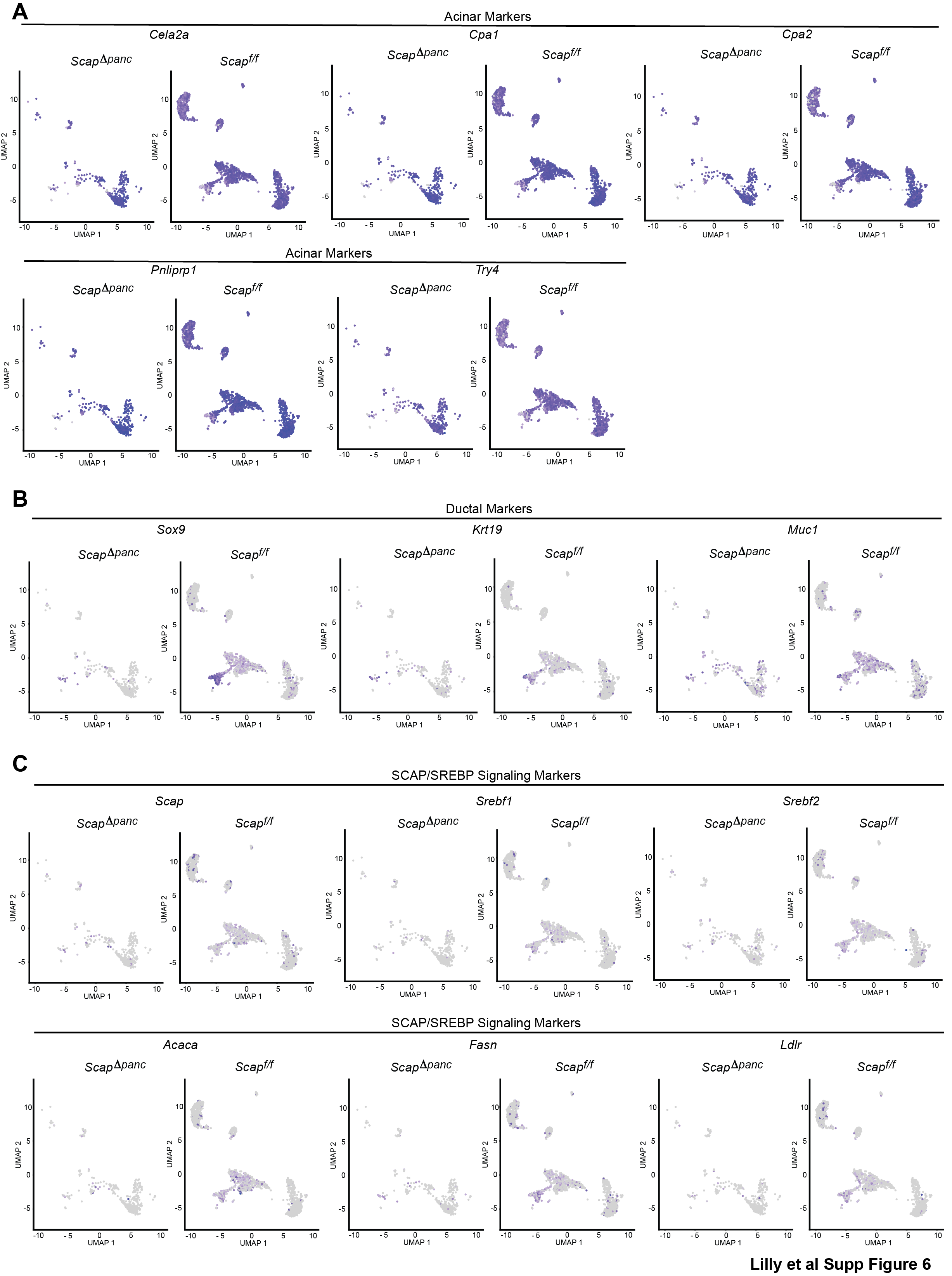


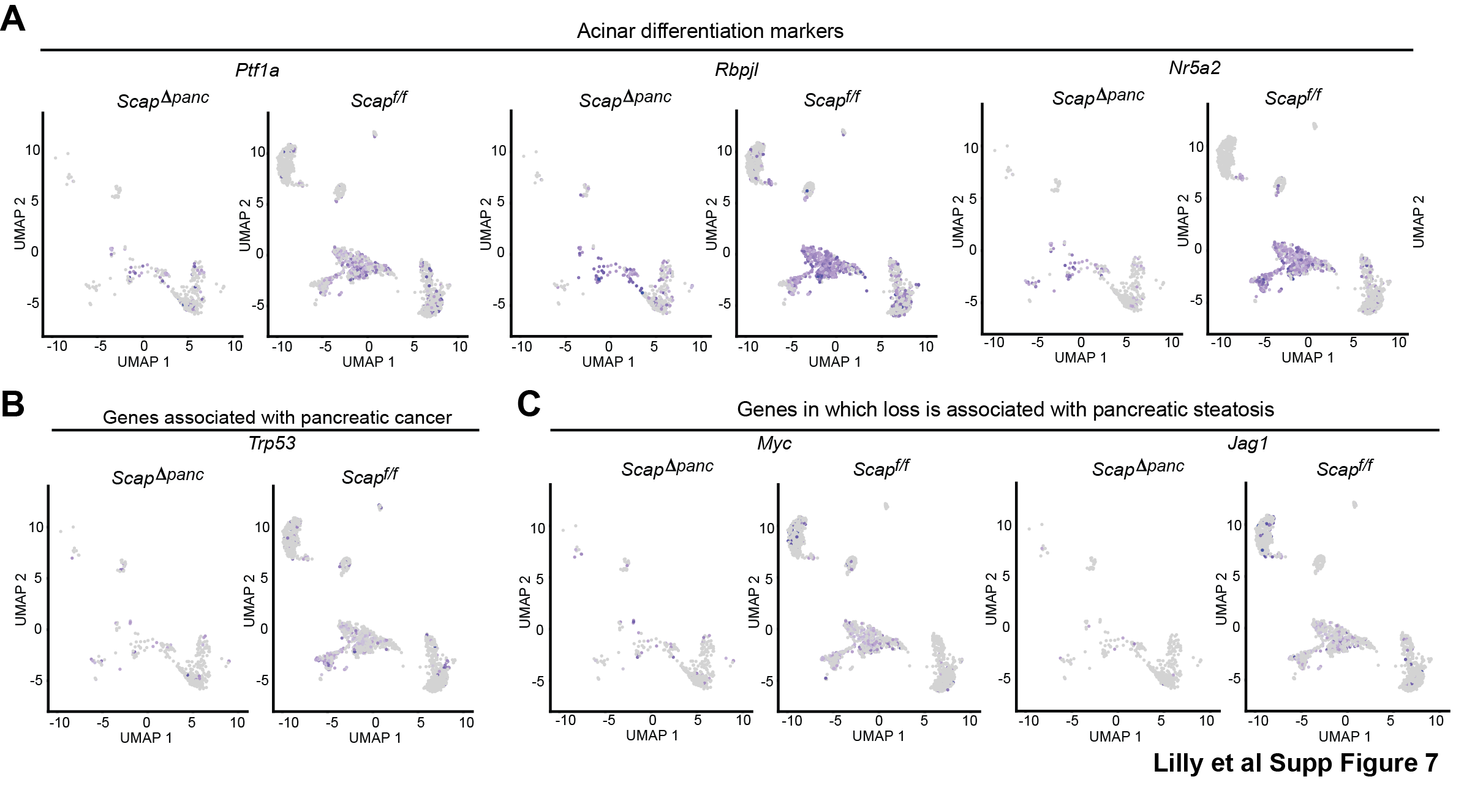


**Supplemental Methods**.

**Mouse models**. All mouse strains were maintained on a C54BL/6J genetic background. All mice were bred and maintained under defined-flora, pathogen-free conditions in rooms monitored and positive for mouse norovirus and Helicobacter spp. at the AAALAC-approved Laboratory Animal Facility at Fox Chase Cancer Center. Age-matched mice of both sexes, equally distributed, were used for experiments unless specified otherwise. All mice were observed and weighed weekly throughout the time of experiments. No randomization was used to determine which animals were allocated to experimental groups. No investigator was blinded to the groups mice were allocated into. Mice with AALAC-specified signs of distress or weight loss of more than 10% were euthanized by CO_2_ inhalation followed cervical dislocation per the Institutional Animal Cancer and Use Committee (IACUC) guidelines at Fox Chase Cancer Center, Philadelphia, PA. Mendelian ratios were calculated by calculating observed genotype ratios of mice from 20-40 litters after which observed genotype ratios were compared to expected genotype ratios per litter using student’s t-test.

**Survival analysis.** After weaning, tumor-bearing mice were monitored every other day by laboratory personnel or staff from the Laboratory Animal Facility at Fox Chase Cancer Center. Mice were euthanized upon exhibiting signs of distress, including >10% body weight loss, hunched posture, lethargy, or significant abdominal distension. Euthanasia was performed by CO₂ inhalation followed by cervical dislocation. Pancreata were processed and evaluated by an experimental pathologist (KQC). Survival curves were generated using the Kaplan–Meier method, and differences between KPC and KPCS mice were assessed using the log-rank (Mantel–Cox) test

**Morphological evaluation.** Pancreata were fixed in 10% phosphate-buffered formaldehyde (formalin) for 24 hours, processed, and embedded in paraffin (FFPE). Sections (5 µm) were cut, deparaffinized, and stained with hematoxylin (Cat. #72604; Epredia) and eosin (Cat. #6766007; Thermo Fisher Scientific) for histologic evaluation (H&E). Slides were scanned using a NanoZoomer S60 Slide Scanner or a Leica Aperio CS2 scanner. Assessment of pancreatic atrophy, acinar-to-ductal metaplasia (ADM), and tumor burden was performed in consultation with an experimental pathologist (KQC). Pancreatic atrophy was scored by an experimental pathologist (KQC) based on evaluation of the entire pancreatic tissue using a standard scale of 0–4, where 0 indicates no atrophy and 4 indicates complete atrophy, as in [2]. The percentage of ADM was determined by an experimental pathologist (KQC) based on whole-pancreas evaluation.

**Cryosection preparation.** For analysis of *wt^Tom^* and *Scap^Δpanc^-Tom* pancreata, tissues were fixed in 4% paraformaldehyde for up to 24 hours at 4°C. Pancreata were then sequentially incubated in 15% sucrose until sinking, followed by incubation in 30% sucrose. Tissues were embedded in optimal cutting temperature (OCT) compound (Cat. #62550; Electron Microscopy Sciences) and stored at −80°C. OCT-embedded pancreata were serially sectioned at 10 µm using a cryostat set to −30°C and stored at −20°C for up to one week prior to staining.

**Immunohistochemical analysis.** Immunohistochemical analysis of protein expression in FFPE tissue was performed using the standard immunohistochemistry protocol from Cell Signaling. Briefly, tissues were deparaffinized and antigen retrieval was performed with citrate retrieval buffer pH 6.0 (Cat#64142-06, Electron Microscopy Sciences, Hatfield, PA). Slides were washed with PBST (PBS 1x + 0.01% Tween-20), incubated in 3% hydrogen peroxide, and blocked for 1 hour with PBS + 5% goatperoxidase (HRP) (Cat#8059, Cell Signaling, Danvers, MA), and slides mounted with Epredia Cytoseal Mountant XYL (Cat#22-050-262, Fisher scientific, Waltham, MA) Samples were imaged and signal quantified using the Vectra 3 Quantitative Pathology Imaging System (Akoya Biosciences, Marlborough, MA, RRID:SCR_025828) and the Aperio CS2 scanner (Leica Biosystems, Nussloch, Germany, RRID:SCR_025111) in the Fox Chase Biosample Repository Core Facility (RRID:SCR_004746). Using these microscopes, whole scans of the slides were captured and used in downstream analysis. Images were analyzed and using Fiji [3], version 2.14.0, National Institute of Health, Bethesda, MD). Immunohistochemical staining of F4/80 was performed on a VENTANA Discovery XT automated staining instrument (Ventana Medical Systems) using VENTANA reagents according to the manufacturer's instructions. The slides were then dehydrated with ethanol series, cleared in xylene, and mounted. Scoring of F4/80 staining was performed by an experimental pathologist (KQC). Quantifications of Ki67 and CD45 was performed using InForm Automated Image Analysis Software (Akoya Biosciences, Marlborough, MA, RRID:SCR_019155).Masson’s trichrome staining was performed using the Masson’s trichrome for connective tissue staining kit (Cat#26367-series, Electron Microscopy Sciences, Hatfield, PA) and the standard staining protocol provided by Electron Microscopy Sciences [4].

Immunofluorescence analysis of FFPE-embedded or frozen tissue sections was performed using standard protocols. FFPE-imbedded sections were deparaffinized and antigen retrieval was performed with citrate retrieval buffer pH 6.0 (Cat#64142-06, Electron Microscopy Sciences, Hatfield, PA). Frozen sections were taken from -20 ^o^C and washed 3 times in PBST to remove excess OCT. All slides were washed with PBST and blocked for 1 hour with PBS + 5% goat serum. After blocking, tissue sections were incubated in primary antibody listed in Supp Table S2. Slides were then washed and incubated in secondary antibody listed in Supp Table S2 as well as DAPI to visualize DNA. Samples were mounted with Vectrashield mounting media (Cat#H-1000-10, Vector Labs, Newark, CA). Samples were imaged using the SP8 advanced confocal system and LASAF (Leica Application Suite Advanced Fluorescence software (Leica Microsystems, Buffalo Grove, IL). For all stainings, 5 representative fields of view were captured per sample. Images were analyzed using Fiji ([3], version 2.14.0, National Institute of Health, Bethesda, MD).

**Single cell and single nuclei RNA isolation**. Post-euthanasia, pancreata were dissected from mice. For tumor bearing mice, pancreata were dissected, snap frozen using liquid nitrogen, and stored at -80^o^C. Once all samples were gathered, single nuclei were extracted and isolated from individual samples using Nuclei Extraction Buffer (Cat#130-128-024, Miltenyi Biotec, San Jose, CA) and Anti-Nucleus MicroBeads (Cat#130-132-997, Miltenyi Biotec, San Jose, CA) according to manufacturer’s instructions. The number of nuclei per sample were determined by counting PI (Cat#550825, BD Pharmingen, San Diego, CA) positive nuclei using ImageXpress Confocal Microscope (Molecular Devices). Samples with a sufficient number of nuclei (>400 nuclei/ul) were used for single nuclei RNA sequencing (snRNA-seq), with a target recovery of 10000 cells. Library construction was performed using the Chromium Next GEM Single Cell 3’ v3.1 dual index gene expression kit (Cat#PN-1000128, PN-1000127, 10x Genomics, Pleasanton, CA) according to the manufacturer’s instructions. The library was processed by Novogene (Sacramento, CA) using the NovaSeq X Plus PE150.

For 2-week-old mice, 5-6 mice pancreata were collected, washed in ice cold Hanks’ Balanced Salt Solution (HBSS), and pooled per genotype (*Scap^Δpanc^* and *Scap^f/f^*), with an equal number of males and females. Pooled pancreata were washed in HBSS, cut into 1-3 millimeter pieces and digested in Dissociation Buffer (1 mg/ml Collagenase P and 1 mg/ml Soybean trypsin inhibitor in HBSS) at 37^o^C for 30 minutes with regular mechanical dissociation of the tissue using a 5 ml pipette. Following tissue disaggregation, 10% FBS was added to halt tissue digestion, and the cell suspensions were filtered through a 100μm strainer (Cat#07-201-432, Thermo Fisher Scientific, Waltham, MA). Samples were spun down at 300 x *g* for 5 minutes at 4^o^C and filtered through a 30μm strainer (Cat#130-098-458, Miltenyi Biotec, San Jose, CA). Dead cells were removed using the dead cell removal kit per manufacture instructions (Cat#130-090-101, Miltenyi Biotec, San Jose, CA). Concentration and viability of collected cells were determined manually using trypan blue staining; Samples with a minimal viability of 90% were used for single cell RNA sequencing (scRNA-seq), with a target recovery of 10000 cells. Library construction was performed using the Chromium Next GEM Single Cell 3’ v3.1 dual index gene expression kit (Cat#PN-1000128, PN-1000127, 10x Genomics, Pleasanton, CA) according to the manufacturer’s instructions. The library was processed by Novogene (Sacramento, CA) using the NovaSeq X Plus PE150.

**Single cell sequencing and analysis.** We aligned the sequence reads to the mm10-2020-A reference genome with default parameters using Cell Ranger 7.1.0 (10x Genomics). The gene matrices were then uploaded into R (version 4.4.1, RRID:SCR_000432) and were analyzed using the Seurat pipeline (version 5.2.1, RRID:SCR_016341). Removal of ambient RNA contamination, quality control, sample normalization and principal component analysis (PCA) were performed using previously described methods [5] and the complete script is available in a Github repository (<https://github.com/lillya349/Scap-project>). Seurat objects were filtered to retail cells with more than 800 unique molecular identifiers (UMIs), less than 10,000 (UMIs), and less than 15% of counts mapped to the mitochondrial genome. Because single cell samples were sequenced and aligned at the same time, they did not require batch correction for downstream analysis. For cluster annotation, each cluster was manually annotated based on differentially expressed genes using the FindMarker function in Seurat. To determine differentially expressed genes (DEG) for gene set enrichment analysis, clusters of interest were subset from all other clusters using the subset() function in Seurat [6]. Subsequently, the FindMarkers() function was used with the Wilcox method, with the log fold change threshold and the minimum percentage of cells set to negative infinite in order to collect all genes in the samples. For downstream analysis, ENSEMBL IDs were added to each differentially expressed gene using the add_ensembl() function generated by our lab and found in the Github repository.

**Single nuclei sequencing and analysis.** Library construction was performed using the Chromium Next GEM Single Cell 3’ v3.1 dual index gene expression kit (Cat#PN-1000128, PN-1000127, 10x Genomics, Pleasanton, CA) according to the manufacturer’s instructions. The library was processed by Novogene (Sacramento, CA) using the NovaSeq X Plus PE150. We aligned the sequence reads to the GRCm39-2024-A reference genome with default parameters using Cell Ranger 8.0.1 (10x Genomics). The gene matrices were then uploaded into R (version 4.4.1) and were analyzed using the Seurat pipeline (version 5.2.1). Removal of ambient RNA contamination, quality control, sample normalization and principal component analysis (PCA) were performed using previously described methods [5] and the complete script is available in a Github repository (<https://github.com/lillya349/Scap-project>). Seurat objects were filtered to retail cells with more than 100 genes, less than 10,000 (UMIs), and less than 30% of counts mapped to the mitochondrial genome. Batch correction was performed to integrate samples sequenced on different days. For cluster annotation, each cluster was manually annotated based on differentially expressed genes using the FindMarker function in Seurat. To determine differentially expressed genes (DEG) for gene set enrichment analysis, clusters of interest were subset from all other clusters using the subset() function in Seurat [6]. Subsequently, the FindMarkers() function was used with the Wilcox method, with the log fold change threshold and the minimum percentage of cells set to negative infinite in order to collect all genes in the samples. For downstream analysis, ENSEMBL IDs were added to each differentially expressed genes using the add_ensembl() function generated by our lab and found in the Github repository.

**Gene set enrichment analysis (GSEA).** The complete script for performing GSEA is available in a Github repository (<https://github.com/lillya349/Scap-project>). Briefly, to perform GSEA from the list of DEG, a pre-ranked list was generated by finding the log_2_(Fold Change)*-log_10_(p val) value for each gene. For the reference gene set, the Hallmark gene sets were taken from the mouse MSigDB collection at the Broad Institute [7, 8]. This reference gene set was then prepared by removing genes not found in our DEG list. The resulting pre-ranked list and reference gene sets were input into fgsea() using the fgsea (version 1.30.0) package with 10,000 permutations and a p-value cut off of less than 0.05. The results were then filtered based on a p adjusted value <0.05, to identify significant relationships to Hallmark gene sets [7, 8].

**Ingenuity pathway analysis (IPA) and Jensen Disease classification.** The output file of all DEGs including Ensembl IDs was input into Ingenuity pathway analysis (Qiagen, Germantown, MD, RRID:SCR_008653), were Ensembl IDs were used as the gene ID for analysis. For the analysis, only genes with a p value of <0.05 were included for IPA Core Analysis. For Jensen Disease analysis, differentially expressed genes generated as previously described were subset to select for genes log(FC) > 2 and p value < 0.05. This gene list was input into Enrichr (<https://maayanlab.cloud/Enrichr/>, RRID:SCR_001575) [9, 10]. The input genelist was then compared to Jensen_DISEASES [11].

**Pseudotime analysis.** The complete script for performing GSEA is available in a Github repository (https://github.com/NDeeSeee/SCAP_scRNA). Briefly, we performed pseudotime trajectory analysis to determine differences in differentiation caused by the loss of Scap. To achieve this, we first subset clusters of interest from all other clusters using the subset() function in Seurat. Using Monocle 3 (version 1.3.7, RRID:SCR_018685) we inferred the pseudotime trajectory of our clusters using a biologically-informed, marker-based root cell selection strategy, drawing on established single‑cell trajectory anchoring methods to select progenitor‑like root cells using composite marker scores; [12], anchored trajectory origins based on gradients of key developmental biomarkers [13], and allowed trajectory orientation by selecting root cells according to expression levels of user‑selected features [14]. We extended these principles by employing multi‑marker early/late expression ratios, adaptive quartile thresholds, z‑score normalization, and deterministic cell selection to ensure statistically robust and biologically grounded trajectory initiation. The markers used are as follows: early markers: acinar and exocrine (*Pdx1, Hnf1b, Sox9, Ptf1a*), fibroblastic (*Vim, Cd44, Vcam1, Pdgfra*), tumor epithelial (*Cdh1, Epcam, Krt8, Krt18*), and tumor fibroblast (*Vim, Des, Acta2*). Late markers: acinar and exocrine (*Amy2a, Cpa1, Prss1, Krt19*), fibroblastic (*Col1a1, Fn1, Acta2, Col6a1*), tumor epithelial (*Vim, Fn1, Snai1, Twist1*), tumor fibroblast (*Pdgfra, Cxcl12, Il6*). The trajectory was then divided into three discrete groups [15].

**Analysis of cell lines**. AR42J cells were generously provided by Dr. Mark Hellmich of the University of Texas Medical Branch, and their identity was confirmed by STR profiling. AR42J cells were cultured in DMEM containing 20% fetal bovine serum (FBS), L-glutamine (L-glu) and penicillin/streptomycin (pen/strep). All cell lines were thawed from frozen stocks and passaged for 3 weeks or less while performing experimental analysis.

To assess consequences of SCAP depletion, *SCAP*-targeting Smartpool siRNA (Cat#SO-2933916G, Horizon Discovery, Lafayette, CO) and scrambled negative control siRNA (Cat#D-001810-10-05, Horizon Discovery, Lafayette, CO) were purchased from Dharmacon. AR42J cells were plated in 6-well plate and transfected with siRNA at a final concentration of 50nM using Dharmafect form 1 (Cat#T-2001-02, Horizon Discovery, Lafayette, CO) according to manufacturer’s instructions. Cells cultured with siRNA transfection for 72 hours and then were harvested for Western Blotting and quantitative RT-PCR.

For Western Blotting, AR42J cells were lysed in Cell-Lytic M lysis buffer (Cat#2978, Sigma-Aldrich, St. Louis, MO) containing Halt Protease Inhibitor Cocktail (#1861278, Thermo Scientific, Waltham, MA). Protein concentrations were quantified using the Pierce BCA Protein Assay Kit (Cat#23225, Thermo Scientific, Waltham, MA). Western blotting was performed using standard procedures. Antibodies and working concentrations used are listed in Supp Table S2. Proteins were visualized using Immobilon Western horseradish peroxidase (HRP) (Cat#WBKLS0500, MilliporeSigma, Burlington MA). Quantification was done using Fiji (PMID: 22743772, version 2.14.0, National Institute of Health, Bethesda, MD) and all values were normalized to relative expression of GAPDH (Cat#97166, Cell Signaling, Danvers, MA).

For qRT-PCR, total RNA was isolated from cells using the Zymo Research Quick-RNA MicroPrep Kit (Cat#R1058, Zymo Research, Irvine, CA) and concentrations were determined using the NanoDrop 1000 spectrophotometer (Thermo Fisher Scientific). Reverse transcription performed as previously described [16], and expression levels of genes of interest were measured using primers listed in Supp Table S1.

**Lipid extraction.** Pancreata were dissected from 2-week-old and 1-month-old *Scap^f/f^* and *Scap^Δpanc^* mice and weighed. Tissues were snap frozen in liquid nitrogen and stored at -80C until samples were processed. Pieces of ~10 mg of frozen tissues were cut on a ceramic tile over dry ice with a new blade kept in dry ice. The tissue was added to low retention Eppendorf tube prepared with 0.6 mL 80% methanol (MeOH) and 20 µL of lipidomics internal standard mix (1:1, SPLASH® LIPIDOMIX #330707: Ceramide/Sphingoid Internal Standard Mixture I #LM6002, both from Avanti Polar Lipids, Alablaster, AL) and kept in dry ice. Samples were pulse sonicated for half-second pulses 30x on ice and kept on ice for 20 min for metabolites extraction. The tissue homogenates were moved to a 10 mL glass Pyrex tube with screw cap. Eppendorf tubes were rinse with 0.5 mL methanol and added to same glass tube. 5 mL methyl tert-butyl ether (MTBE) was added to each of the tubes and then tubes were shaken vigorously for 30 min. 1.2 mL water was added to each tube and vortexed for 30 sec each. Samples were centrifugated for 10 min @ 1000 x *g* to separate two phases. The top clarified phase was moved to a clean glass Pyrex tube and evaporated to dryness under nitrogen. 200 µL MTBE/MeOH=1/3 (v/v) per 10 mg tissue was used to re-suspend the residue. The sample was spun down at 10, 000 x *g* for 10 min at 4°C and the top 100 µL were transferred to a HPLC vial for LC-MS analysis. A pooled sample was created by mixing 20 µL of each re-suspended sample and ran as quality control (QC) every 15 samples. 2 µL injections were made in both positive mode and separately in the negative mode.

**Liquid chromatography-high resolution mass spectrometry (LC-HRMS) for lipids.** Lipid analysis on the extracted samples was conducted by LC-HRMS as previously described [17]. Briefly, separations were conducted at 55 °C on an Ultimate 3000 (Thermo Fisher Scientific, Waltham, MA) using an Ascentis Express C18, 2.1 × 150 mm 2.7μm column (Sigma-Aldrich, St. Louis, MO). The flow rate was 0.4 m /min, solvent A was water:acetonitrile (4:6 v/v) with 0.1% formic acid and 10 mM ammonium formate and solvent B was acetonitrile:isopropanol (1:9 v/v) with 0.1% formic acid and 10 mM ammonium formate. The gradient was as follows: 1 % B at 0 min, 10% B at 1 min, 40% B at 4 min, 75% B at 12 min, 99% B at 21 min, 99% B at 24 min, 10% B at 24.5 min, 10% at 30 min.

For HRMS analysis, a QE Exactive-HF mass spectrometer (Thermo Fisher Scientific, Waltham, MA) calibrated within 3 days prior was used in positive ion mode with an HESI source. The operating conditions were: spray voltage at 3.5 kV; capillary temperature at 285°C; auxiliary temperature 370°C; tube lens 45. Nitrogen was used as the sheath gas at 45 units, the auxiliary gas at 10 units and sweep gas was 2 units. Same MS conditions were used in negative ionization mode, but with a spray voltage at 3.2 kV. Control extraction blanks were made in the same way using just the solvents instead of the tissue homogenate. The control blanks were used for the exclusion list with a threshold feature intensity set at 1e10^^5^. Untargeted analysis and targeted peak integration was conducted using LipidsSearch 4.2 (Thermo Fisher Scientific, Waltham, MA) as described [18]. All samples were analyzed in a randomized order in full scan MS that alternated with MS2 of top 20, with HCD scans at 30, 45 or 60 eV. Full scan resolution was set to 120,000 in the scan range between *m/z* 250–1800. The pool sample was run every 15 samples. Lipids quantification was done from the full scan data. The areas were normalized based on the amount of the internal standard added for each class. All amounts were normalized to the original tissue weight.

**Lipidomic Analysis.** Additional analysis was performed using MetaboAnalyst 6.0 ([19], RRID:SCR_015539). Using the web browser version of MetaboAnalyst 6.0, samples were analyzed using the Statistical Analysis [one factor] function. No additional quality control (QC) filtering or data normalization was performed on the samples, as this step was integrated in lipid annotation during Lipid Search previously. When performing the analysis, a fold change (FC) threshold of 2.0 and p-value threshold of 0.05 was used to generate volcano plots and interpret its results.

**Data availability statement.** The data generated in this study are available within the article and its supplementary data files. The sequence data generated in this study are publicly available in Gene Expression Omnibus (GEO) at GSE306231 and GSE317919.

**References**

1 Posta M, Gyorffy B. Analysis of a large cohort of pancreatic cancer transcriptomic profiles to reveal the strongest prognostic factors. *Clin Transl Sci* 2023; 16: 1479-1491.

2 Niazi AA, Kourkinejad Gharaei F, Saebinasab Z, Maleki M, Maghool F, Fereidooni F *et al*. Eugenol Administration Improves Liver Damage Induced by a Fructose-Rich Diet. *Adv Biomed Res* 2021; 10: 42.

3 Schindelin J, Arganda-Carreras I, Frise E, Kaynig V, Longair M, Pietzsch T *et al*. Fiji: an open-source platform for biological-image analysis. *Nature methods* 2012; 9: 676-682.

4 Sheehan DC, Hrapchak BB. *Theory and practice of histotechnology, Second Edition*. Battelle Pr: St. Louis, 1987.

5 Carpenter ES, Elhossiny AM, Kadiyala P, Li J, McGue J, Griffith BD *et al*. Analysis of Donor Pancreata Defines the Transcriptomic Signature and Microenvironment of Early Neoplastic Lesions. *Cancer Discov* 2023; 13: 1324-1345.

6 Satija R, Farrell JA, Gennert D, Schier AF, Regev A. Spatial reconstruction of single-cell gene expression data. *Nat Biotechnol* 2015; 33: 495-502.

7 Liberzon A, Birger C, Thorvaldsdottir H, Ghandi M, Mesirov JP, Tamayo P. The Molecular Signatures Database (MSigDB) hallmark gene set collection. *Cell Syst* 2015; 1: 417-425.

8 Castanza AS, Recla JM, Eby D, Thorvaldsdottir H, Bult CJ, Mesirov JP. Extending support for mouse data in the Molecular Signatures Database (MSigDB). *Nature methods* 2023; 20: 1619-1620.

9 Chen EY, Tan CM, Kou Y, Duan Q, Wang Z, Meirelles GV *et al*. Enrichr: interactive and collaborative HTML5 gene list enrichment analysis tool. *BMC bioinformatics* 2013; 14: 128.

10 Kuleshov MV, Jones MR, Rouillard AD, Fernandez NF, Duan Q, Wang Z *et al*. Enrichr: a comprehensive gene set enrichment analysis web server 2016 update. *Nucleic Acids Res* 2016; 44: W90-97.

11 Pletscher-Frankild S, Palleja A, Tsafou K, Binder JX, Jensen LJ. DISEASES: text mining and data integration of disease-gene associations. *Methods* 2015; 74: 83-89.

12 Sumanaweera D, Suo C, Cujba AM, Muraro D, Dann E, Polanski K *et al*. Gene-level alignment of single-cell trajectories. *Nature methods* 2025; 22: 68-81.

13 Chen H, Albergante L, Hsu JY, Lareau CA, Lo Bosco G, Guan J *et al*. Single-cell trajectories reconstruction, exploration and mapping of omics data with STREAM. *Nat Commun* 2019; 10: 1903.

14 Faure L, Soldatov R, Kharchenko PV, Adameyko I. scFates: a scalable python package for advanced pseudotime and bifurcation analysis from single-cell data. *Bioinformatics* 2023; 39.

15 Tian C, Zhang Y, Tong Y, Kock KH, Sim DY, Liu F *et al*. Single-cell RNA sequencing of peripheral blood links cell-type-specific regulation of splicing to autoimmune and inflammatory diseases. *Nat Genet* 2024; 56: 2739-2752.

16 Deneka AY, Kopp MC, Nikonova AS, Gaponova AV, Kiseleva AA, Hensley HH *et al*. Nedd9 Restrains Autophagy to Limit Growth of Early Stage Non-Small Cell Lung Cancer. *Cancer Res* 2021; 81: 3717-3726.

17 Wang D, Ho ES, Cotticelli MG, Xu P, Napierala JS, Hauser LA *et al*. Skin fibroblast metabolomic profiling reveals that lipid dysfunction predicts the severity of Friedreich's ataxia. *J Lipid Res* 2022; 63: 100255.

18 Wang D, Xu P, Mesaros C. Analytical considerations for reducing the matrix effect for the sphingolipidome quantification in whole blood. *Bioanalysis* 2021; 13: 1037-1049.

19 Pang Z, Lu Y, Zhou G, Hui F, Xu L, Viau C *et al*. MetaboAnalyst 6.0: towards a unified platform for metabolomics data processing, analysis and interpretation. *Nucleic Acids Res* 2024; 52: W398-W406.
